# Genomes of historical specimens reveal multiple invasions of LTR retrotransposons in *Drosophila melanogaster* during the 19th century

**DOI:** 10.1073/pnas.2313866121

**Published:** 2024-04-02

**Authors:** Almorò Scarpa, Riccardo Pianezza, Filip Wierzbicki, Robert Kofler

**Affiliations:** ^a^Institut für Populationsgenetik, Vetmeduni Vienna, Wien 1210, Austria; ^b^Vienna Graduate School of Population Genetics, Vetmeduni Vienna, Vienna 1210, Austria

**Keywords:** transposable elements, *Drosophila melanogaster*, population genetics, LTR retrotransposon, genome evolution

## Abstract

Transposable element invasions have a profound impact on the evolution of genomes and phenotypes. It is thus an important open question how often such TE invasions occur. To address this question, we utilize the genomes of historical specimens, sampled about 200 y ago. We found that the LTR retrotransposons Blood, Opus, and 412 spread in *Drosophila melanogaster* in the 19th century. These invasions constitute second waves, as degraded fragments were found for all three TEs. The composition of Opus and 412, but not of Blood, shows a pronounced geographic heterogeneity, likely due to founder effects during the invasions. Finally, we identified species from the *Drosophila simulans* complex as the likely origin of the TEs. We show that in total, seven TE families invaded *D. melanogaster* during the last 200y, thereby increasing the genome size by up to 1.2Mbp. We suggest that this high rate of TE invasions was likely triggered by human activity. Based on the analysis of strains and specimens sampled at different times, we provide a detailed timeline of TE invasions, making *D. melanogaster* the first organism where the invasion history of TEs during the last two centuries could be inferred.

## Significance

Transposable elements (TEs) are stretches of selfish DNA that have a massive impact on evolution. Using the genomes of old fruit flies, maintained for about 200 y in museum collections, we show that seven TEs spread in the fly genome during the last two centuries. We argue that such a high rate of TE invasions is highly unusual and propose that human activity, contributing to habitat expansions of species and thus amplifying the opportunities for horizontal transfer of these selfish elements, is likely responsible for these TE invasions.

Transposable elements (TEs) are short stretches of DNA that selfishly multiply within genomes. These elements can be broadly classified into two main classes: class I, also known as retrotransposons, and class II, referred to as DNA transposons. Retrotransposons propagate via a copy-and-paste mechanism that involves an RNA intermediate as a template, whereas DNA transposons directly relocate to new genomic locations using a cut-and-paste mechanism (1–3). For retrotransposons, usually long-terminal repeat (LTR) and non-LTR TEs can be distinguished (1, 2, 4). TEs are highly successful, having invaded virtually all eukaryotic species investigated so far (2).

Since many TE insertions are likely deleterious (5, 6), host organisms have evolved elaborate defense mechanisms against them (7–9). In *Drosophila melanogaster*, the defense against TEs is based on piRNAs (PIWI-interacting RNAs), i.e., small RNAs with a size between 23 and 29 nt, that repress TE activity at the transcriptional and posttranscriptional level (7, 10–12). These piRNAs are largely derived from discrete genomic loci, the piRNA clusters (7). In *D. melanogaster*, piRNA clusters account for about 3% of the genome (7). It is thought that a TE invasion is stopped when a copy of the invading TE jumps into a piRNA cluster, which triggers the emergence of piRNAs complementary to the invading TE (13–18). One particularly important component of the piRNA pathway is the ping-pong cycle, which amplifies the piRNAs by alternately cleaving sense and antisense transcripts of TEs (7, 10). Activation of the ping-pong cycle may be necessary for silencing an invading TE (19). Once a TE is inactivated, all insertions of a family will decay by accumulating mutations over time. Eventually, the TE might not be able to mobilize anymore, resulting in the death of a TE family (20). One strategy for escaping this inactivation, thus ensuring the long-term persistence of a TE, is horizontal transfer (HT) to a naive species not having the TE (20). Such HT may trigger TE invasions in the naive species, that are in turn silenced by the host defense (20). HT is probably abundant. For example, a study investigating 195 insect species identified about 2,000 HTs of TEs (21). Multiple HT of TEs were also reported in *D. melanogaster* (22, 23). In agreement with this, most LTR families in *D. melanogaster* are likely of recent origin, possibly as young as 16,000y (24, 25).

Furthermore, four different TEs invaded *D. melanogaster* populations during the last 100y (26). Three of these TE invasions—the P-element, Hobo, and the I-element—were discovered due to phenotypic effects caused by the activity of the TE, i.e., the hybrid dysgenesis (HD) symptoms (27–34). Crosses between males having a TE and females not having it frequently lead to diverse phenotypic effects, such as atrophied ovaries, whereas no phenotypic effects could be found in reciprocal crosses (27). By sequencing some of the oldest available *Drosophila* strains, we recently found that a fourth TE, i.e., Tirant, also invaded *D. melanogaster* populations during the last century (26). We did not notice any HD symptoms caused by Tirant, which may account for the late discovery of the Tirant invasion (26). Hobo, the I-element, and Tirant likely spread in *D. melanogaster* in multiple waves as degraded and fragmented copies of these TEs could be found in all investigated strains (26). Solely the P-element did not show similarity to any sequence of the naive *D. melanogaster* genome. Interestingly, we found that the Tirant composition showed geographic heterogeneity where populations from Tasmania carried slightly different Tirant variants than other populations, likely due to a founder effect during the invasion (26). In agreement with such a geographic heterogeneity of the TE composition, a recent work identified diverse TE lineages (i.e., SNPs showing correlated allele frequencies across different samples) for multiple TE families in *D. melanogaster* (35).

Based on *D. melanogaster* strains sampled at different time points, previous works suggested that the Tirant and the I-element invasions occurred between 1930 and 1950, the Hobo invasion around 1955, and the P-element invasion between 1950 and 1980 (26, 28–30). The invasion history could only be reconstructed up to ≈1930 as the oldest available lab strains, Oregon-R and Canton-S, were sampled between 1925 and 1935 (26). Recently, the genomes of 25 historical *D. melanogaster* specimens became publicly available which provides us with an opportunity to extend the invasions history of *D. melanogaster* by another 100y (36). Six strains were sampled around 1800 in Lund (Sweden; early 1800), two around 1850 in Passau (Germany; mid-1800), one around 1900 in Lund (late 1800), and 16 around 1933 in Lund (36). By analyzing the genomes of these historical specimens, we found that three transposons—Blood, Opus, and 412—invaded *D. melanogaster* populations likely between 1850 and 1933. All three TEs are LTR retrotransposons. Blood and 412 lack an envelope protein and belong to the Gypsy/mdg1 superfamily. Opus (also known as Nomad) possesses an envelope protein and is classified under the Gypsy/Gypsy superfamily (4). Similarly to Tirant (Gypsy/Gypsy superfamily), Opus may thus form virus-like-particles that could infect the germline. All three TEs have a similar size (Blood=7,410bp, 412=7,567bp, and Opus=7,512bp), between 2 and 4 annotated ORFs (Blood=3,412=4, and Opus=2), and LTRs with a similar size (Blood=398bp, 412=514bp, and Opus=518bp) (37). We also found remnants of previous invasions for all three TEs. By investigating TE-specific SNPs in extant populations, we found that the composition of Opus and 412—but not of Blood—varies among populations, where especially populations from Zimbabwe carry slightly different variants than other populations. This geographic heterogeneity could be due to founder effects during the invasion of Opus and 412. We suggest that HT from a species of the *Drosophila simulans* complex likely triggered the invasions of Blood, Opus, and 412. By jointly analyzing the genomes of strains and specimens sampled at different times, we extend the invasion history of TEs in *D. melanogaster* by another 100y: Blood, Opus, and 412 invaded between 1850 and 1933, followed by Tirant and the I-element between 1930 and 1950, Hobo around 1955, and finally the P-element between 1950 and 1980 (see also ref. 26). To our knowledge, this makes *D. melanogaster* the first species where it is feasible to infer a detailed invasion history of TEs during the last two centuries.

## Results

### The LTR Retrotransposons Blood, 412, and Opus Likely Invaded D. melanogaster Populations between 1800 and 1933.

**Fig. 1.**
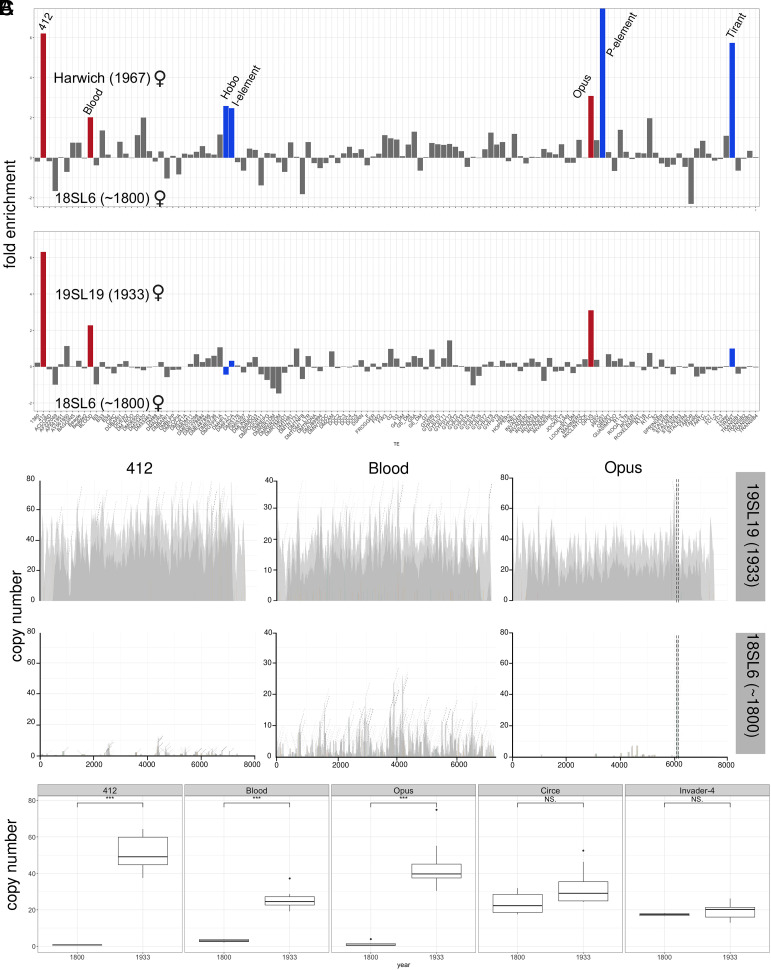
Genomes of historical *D. melanogaster* specimens suggest that the LTR retrotransposons Opus, Blood, and 412 invaded *D. melanogaster* populations in the 19th century. (*A*) Differences in TE abundance between a strain collected around 1800 (18SL6) and strains collected in 1933 (19SL19) and 1967 (Harwich). For each TE family (*x*-axis), we show the fold enrichment estimated with DeviaTE (*y*-axis). Blue bars represent previously identified TE invasions, while red bars indicate new ones. (*B*) Abundance and diversity of 412, Blood, and Opus in a strain collected around 1800 and 1938. The short reads (100bp) were aligned to the consensus sequences of these TEs and visualized with DeviaTE. The normalized coverage (using three single-copy genes) provides a proxy for the copy number of a TE. SNPs and indels are shown as colored lines. Coverage based on unambiguously and ambiguously aligned reads is shown in dark and light gray, respectively. For Opus, the coverage was manually curbed at the poly-A track (dashed lines). (*C*) Copy numbers of 412, Opus, and Blood in historical specimens collected around 1800 (9 samples) and 1933 (16 samples). As controls, Circe and Invader-4 are included. The significance was computed with Wilcoxon rank-sum tests.

Sequencing of the oldest available *D. melanogaster* strains, sampled between 1925 and 1938, revealed invasions of four different TEs (Tirant, Hobo, I-element, and P-element) in natural populations during the last 100y (26). The publication of the genomes of 25 historical *D. melanogaster* specimens, collected between 1800 and 1933, provides us with the opportunity to investigate whether additional TE invasion occurred between 1800 and 1933 (36). To do this, we compared the abundance of TEs in the historical specimens to more recently collected strains. We downloaded the publicly available reads, filtered or trimmed them to a size of 100bp, aligned the reads to the consensus sequences of TEs in *D. melanogaster* (37), and estimated the abundance of TEs with our tool DeviaTE (38) (for an overview of the data used in this study, see *SI Appendix*, Table S1). For each TE family, DeviaTE normalizes the average coverage of a TE (e.g., 121) to the average coverage of single-copy genes (e.g., 12), which allows inferring the TE copy number per haploid genome (e.g., 10.1=121/12; the average coverage is also used for samples with a heterogeneous coverage). We first compared the TE abundance between a strain collected around 1800 (18SL6) and the strain Harwich. Harwich was collected around 1967 and should thus contain copies of all TEs that invaded *D. melanogaster* populations during the last 100y [i.e., Tirant, Hobo, I-element, and P-element; (26)]. As expected, we found a strong overrepresentation of Tirant, Hobo, the I-element, and the P-element in Harwich (Fig. 1*A*, blue). Surprisingly, we additionally found that 412, Blood, and Opus are highly overrepresented in Harwich as compared to 182L6 (Fig. 1*A*, red). A comparison between 182L6 and a strain collected around 1933 (19SL19) showed an overrepresentation of 412, Blood, and Opus in 19SL19 but not of the I-element, Hobo, Tirant, and the P-element (Fig. 1*A*, red). By contrast, a comparison between 19SL19 and Harwich solely revealed an overrepresentation of Tirant, the I-element, Hobo, and the P-element but not of Opus, Blood, and 412 (*SI Appendix*, Fig. S1).

An analysis of the coverage showed that 412, Blood, and Opus have a uniformly elevated coverage in 19SL19 (1933) as compared to 18SL6 (1800; Fig. 1*B*), which suggests that overrepresentation of these three TEs in Harwich is not an alignment artifact (e.g., due to low complexity regions). Such a uniformly elevated coverage in specimens sampled around 1933 as compared to those sampled around 1800 can be found for all of the analyzed samples (*SI Appendix*, Figs. S2–S4). Comparing the sequences of these three TEs with BLAST did not reveal any sequence similarity, ruling out cross-mapping among these three TEs. Solely a few high-frequency SNPs can be found for the three TEs in Harwich, which suggests that most of the reads align without mismatch to the consensus sequence to the TEs (Fig. 1*B*). By contrast, only few highly diverged reads align to Opus, 412, and Blood in 18SL6 (Fig. 1*B*). The estimated copy numbers of 412, Opus, and Blood are significantly lower in specimens collected around 1800 as compared to specimens collected at 1933, whereas no significant differences could be found for other TEs such as Circe and Invader4 (Fig. 1*C*). Out of 150 TE families, solely Blood, Opus, and 412 have significantly elevated copy numbers in specimens collected at 1933 compared to specimens collected between 1800 and 1875 (*SI Appendix*, Fig. S5). An analysis independent of DeviaTE, solely based on the number of reads aligning to the TEs, confirms this significant difference in the abundance of 412, Blood, and Opus when comparing strains sampled in 1800 and 1933 (*SI Appendix*, Fig. S6). So far, we solely considered reads with a length of at least 100bp. However, most of the reads from historical samples are degraded with a length of 50bp (36). We thus repeated these analyses with reads of 50bp (longer reads were trimmed) and again found significantly elevated copy number differences for Blood, Opus, and 412 but not for Circe and Invader-4 between strains collected around 1800 and 1933 (*SI Appendix*, Fig. S7). Our data thus suggest that 412, Blood, and Opus invaded natural *D. melanogaster* populations between 1800 and 1933. To further test this hypothesis, we investigated the length and divergence of these TEs in four high-quality assemblies (mostly based on long reads) of *D. melanogaster* [Canton-S, Iso1, Pi2, and Dgrp-732 (39–41)]. For recently active TEs we expect to find multiple full-length insertions with a high similarity to the consensus sequence. Indeed, in each analyzed strain, we found multiple full-length insertions of Blood, Opus, and 412 that showed little divergence to the consensus sequence (<1%; *SI Appendix*, Fig. S8). For Opus and 412, we additionally found some highly diverged (20 to 30%) fragments close to the ends of the chromosome arms (likely heterochromatin; *SI Appendix*, Fig S9). When these diverged fragments are included in our analysis pipeline, solely few ambiguously aligned reads mapped to the consensus sequence of the TEs (*SI Appendix*, Figs. S2 and S4), suggesting that the diverged fragments account for the few reads mapping to Opus and 412 in the historical samples. Therefore, full-length insertions of Opus and 412 were likely absent in *D. melanogaster* around 1800. For Blood, the situation is slightly more complex. We found four full-length insertions of sequences resembling Blood (2% divergence from the consensus sequence on the average) close to heterochromatic regions (*SI Appendix*, Results 1). The LTRs of these four Blood insertions are quite distinct from the LTRs of the consensus Blood (25% divergence). We estimate that these four insertions are about 650,000y old and show that they may account for the continuous coverage of Blood in samples collected around 1800 (*SI Appendix*, Results 1).

In summary, we suggest that the LTR retrotransposons Blood, Opus, and 412 invaded natural *D. melanogaster* populations in the 19th century. These recent invasions likely constitute second waves of invasions, as we found degraded fragments of these TEs in all investigated strains.

### The Invasion History of TEs in D. melanogaster during the Last 200y.

**Fig. 2.**
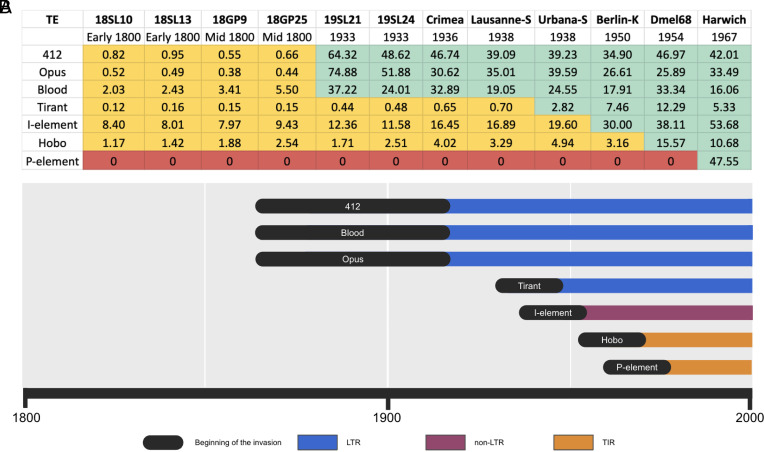
History of TE invasions in *D. melanogaster* during the last 200y. (*A*) Overview of the abundance of Blood, Opus, 412, Tirant, the I-element, Hobo, and the P-element in strains or specimens sampled at different years. For each TE family, we classified the abundance into the following categories: absence of TE (red), presence of solely degraded copies (likely remnants of ancient invasions; yellow), and presence of non-degraded copies with a high similarity to the consensus sequence (green). The numbers represent copy numbers in haploid genomes as estimated by DeviaTE. (*B*) Timeline of TE invasions in *D. melanogaster*. The width of the black bars indicates the range of uncertainty of the invasions.

In a previous work, we inferred the history of TE invasions in natural *D. melanogaster* populations by sequencing different laboratory strains collected during the last century (26). The oldest available strains, Oregon-R and Canton-S, were collected around 1925 to 1936. Given the availability of the museum specimens, we aim to extend this work by another 100y, thus inferring the invasion history of TEs in *D. melanogaster* during the last 200y (until 1800). We estimated the copy numbers of the seven TEs that recently invaded *D. melanogaster* (Blood, Opus, 412, Tirant, the I-element, hobo, and the P-element) in the historical specimens as well as in diverse strains sampled during the last century (for an overview of all investigated strains and specimens, see *SI Appendix*, Table S1). We trimmed reads to a size of 100bp, mapped them to the consensus sequences of TEs in *D. melanogaster*, and estimated the copy numbers with DeviaTE [see above; (38)]. Opus, Blood, and 412 were largely absent in all strains sampled until ≈1850 (Fig. 2*A* and *SI Appendix*, Table S1). We noticed a sudden increase in the number of reads mapping to Opus, Blood, and 412 starting in some samples collected in the late 1800s, where these three TEs were present in all specimens collected after 1933 (Fig. 2*A* and *SI Appendix*, Table S1; for examples of our classification of the TE abundance, see *SI Appendix*, Figs. S2–S4 and S10). We thus suggest that Opus, Blood, and 412 invaded natural *D. melanogaster* populations between 1850 and 1933 (Fig. 2*B* and *SI Appendix*, Table S1). To provide the complete invasion history of TEs during the last 200y, we also estimated the abundance of the TE families which invaded *D. melanogaster* during the last century, i.e., Tirant, Hobo, the I-element, and the P-element (26). In agreement with previous works, our data suggest that Tirant invaded *D. melanogaster* populations between 1933 and 1950, followed by the I-element, Hobo, and finally by the P-element [Fig. 2*B* and *SI Appendix*, Table S1 (26, 28–30)]. In summary, we suggest that the LTR retrotransposons Opus, Blood, and 412 invaded natural *D. melanogaster* populations between ≈1850 and 1933, Tirant and the I-element between 1933 and 1950, Hobo around 1955, and the P-element between 1950 and 1980. To our knowledge, *D. melanogaster* is the first species where the history of TE invasions during the last centuries could be inferred.

### Blood, Opus, and 412 Are Silenced by the piRNA Pathway in Natural Populations.

We next asked whether Blood, Opus, and 412 are under host control by the piRNA pathway in extant populations. To address this question, we interrogated small RNA data from the Global Diversity Lines (GDL) which comprise 85 *D. melanogaster* strains sampled after 1988 from five different continents (Africa—Zimbabwe, Asia—Beijing, Australia—Tasmania, Europe—Netherlands, and America—Ithaca (42). The small RNA were sequenced for 10 out of the 85 GDL strains, where two strains were selected from each continent (43). We found abundant sense and antisense piRNAs distributed over the entire sequence of the three TEs in all 10 GDL strains (*SI Appendix*, Fig. S11). In the germline, the amount of piRNAs complementary to a TE is greatly amplified by the ping-pong cycle (7, 10). The activity of this ping-pong cycle is likely necessary to establish host control over an invading TE (19, 44). An active ping-pong cycle generates a characteristic overlap between the 5^′^ positions of sense and antisense piRNAs, i.e., the ping-pong signature (7, 10). All three TEs show noticeable ping-pong signatures in the 10 analyzed GDL strains (*SI Appendix*, Fig. S12). We thus argue that Blood, Opus, and 412 are controlled by the piRNA pathway in extant *D. melanogaster* populations.

### The Composition of Opus and 412 but Not of Blood Varies Among Extant Populations.

**Fig. 3.**
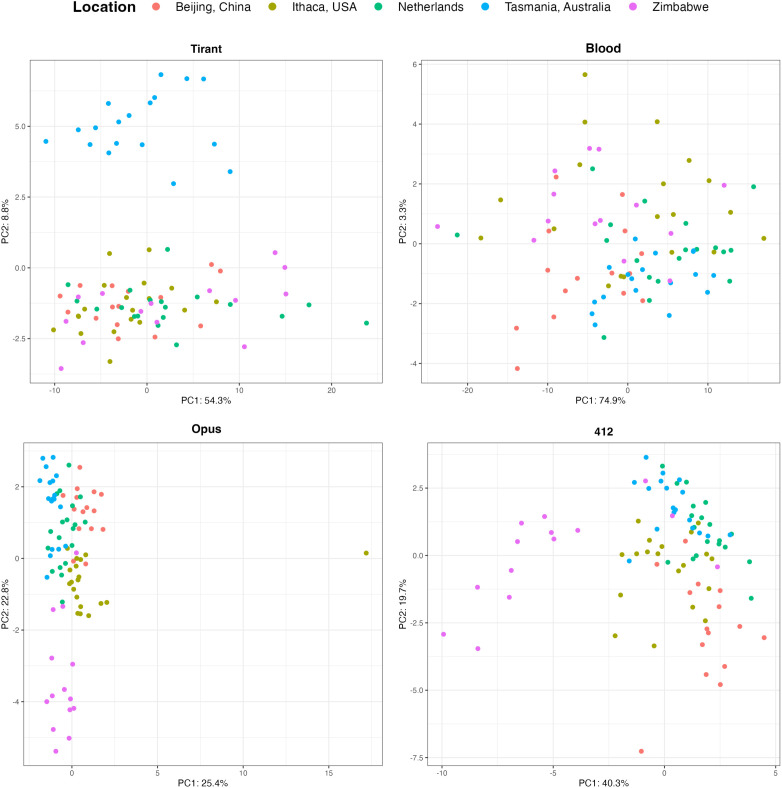
PCAs for Tirant, Blood, Opus, and 412 in the 85 GDL strains. PCAs are based on the allele frequency of TE-specific SNPs. Note that Tirant, Opus, and 412 show population structure, likely due to founder effects during the invasion.

We previously found that the Tirant composition varies among populations, where especially populations from Tasmania carried different variants than populations from other geographic locations (26). To investigate whether a geographic heterogeneous composition can also be found for Blood, Opus, and 412, we analyzed the composition of these TEs in the 85 GDL strains (42); for an overview of all analyzed strains, see *SI Appendix*, Table S2]. For each TE family, we identified SNPs and estimated the allele frequencies of the SNPs. Notably, in this work, a SNP refers to a variant among dispersed TE copies. Our allele frequency estimates thus reflect the TE composition within a particular strain (e.g., if 15 Blood insertions in a strain carry a “G” at a particular site and 5 an “A,” the frequency of G at this site is 0.75). We used PCA to summarize differences in the TE composition among the GDL strains. We first confirmed that PCAs capture the previously reported geographic heterogeneity of Tirant [Fig. 3; (26)]. We further found that Opus and 412, but not Blood, show geographic heterogeneous compositions (Fig. 3). For Opus, populations from Tasmania and Zimbabwe show distinct clusters, while for 412, populations from Zimbabwe, and to a minor extent from Beijing, form separate clusters. To rule out that these geographic patterns are merely due to the ancient fragments of these TEs, we repeated these analyses by excluding all sites having a coverage in specimens collected around 1800, but we found the same clusters in the PCA (*SI Appendix*, Fig. S13). We next investigated the reasons for these distinct clusters in the PCA. Therefore, we aimed to identify diagnostic SNPs for these TEs, i.e., SNPs that are abundant in a population of interest but rare in all other populations (*SI Appendix*, Fig. S14). We found several diagnostic SNPs with a high frequency for Tirant in Tasmania; Opus in Zimbabwe and Tasmania; and 412 in Zimbabwe and Beijing (*SI Appendix*, Fig. S14). No diagnostic SNPs with a high frequency were found for Blood (*SI Appendix*, Fig. S14). Removing these diagnostic SNPs led to a collapse of the geographic clustering in the PCA (*SI Appendix*, Fig. S15). We thus argue that the diagnostic SNPs are responsible for the clusters of the PCA. For an overview of the most distinct diagnostic SNPs, see *SI Appendix*, Table S3. Differences in the TE composition among the GDL populations are thus responsible for the geographic heterogeneity observed for Tirant, Opus, and 412. Interestingly, the geographic clusters seen for 412 resemble the pattern observed with “neutral” autosomal SNPs, where populations from Zimbabwe and Beijing also form distinct clusters from the other populations (42). The geographic heterogeneity in the TE composition could be either due to founder effects during the TE invasions or to demographic processes (*Discussion*).

### Origin of Horizontal Transfer.

**Fig. 4.**
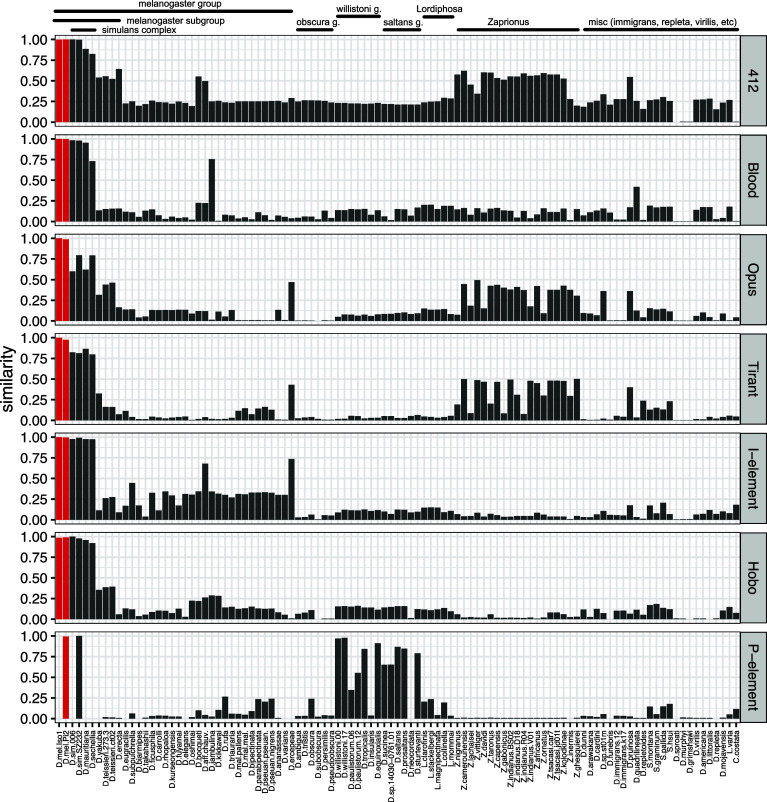
Possible origins of the seven TE families that invaded *D. melanogaster* populations during the last 200y. Data are shown for 103 long-read assemblies of diverse drosophilid species (red *D. melanogaster*). The barplots show the similarity of TE insertions in a given assembly to the *D. melanogaster* consensus sequence of the TE family. For example, a value of 0.9 indicates that at least one TE insertion in the assembly has a high similarity (≈90%) to the consensus sequence of the TE. Apart from the P-element, which was likely transmitted from *Drosophila willistoni* to *D. melanogaster*, all other TE families have insertions with the highest similarity in species from the *D. simulans* complex. HT of insertions from the *D. simulans* complex thus likely triggered the invasions of Blood, Opus, and 412 in *D. melanogaster* populations.

Here, we propose that Blood, Opus, and 412 recently invaded *D. melanogaster* populations, likely following horizontal transfer (HT) from a different species. To identify the possible source of the HT, we investigated the genomes of 101 long-read assemblies of different drosophilid species (45). We also included the long-read assemblies of recently collected *D. melanogaster* and *D. simulans* strains [Pi2, SZ232 (46, 47)]. We reasoned that the species that acted as donor for HT should have insertions with a high similarity to the consensus sequence of the TEs in *D. melanogaster*. Using RepeatMasker, we identified TE insertions in these 103 assemblies and estimated the similarity of the insertions to the consensus sequence of *D. melanogaster*. We first tested whether this approach allows us to reproduce the likely donor species for Tirant, I-element, P-element, and Hobo. Apart from *D. simulans*, which recently acquired the P-element (48, 49), we find that *D. willistoni* carries P-element insertions that are most similar to the *D. melanogaster* P-element (Fig. 4). This is in agreement with previous work suggesting that a species from the willistoni or saltans group is the likely source of the P-element in *D. melanogaster* (32). For Hobo, the I-element, and Tirant, a species from the *D. simulans* complex was suggested as the likely donor (26, 31, 50, 51). In agreement with this, we also find that species from the *D. simulans* complex have insertions that are most similar to the consensus sequence of Tirant, Hobo, and the I-element (Fig. 4). Interestingly, we found that species from the *D. simulans* complex have insertions that are most similar to the consensus sequence of Blood, Opus, and 412 (Fig. 4). One problem with this analysis is that we cannot infer the direction of the HT. It is, for example, possible that an HT from *D. melanogaster* triggered invasions of Blood, Opus, and 412 in species of the *D. simulans* complex and that the invasions of these TEs in *D. melanogaster* were actually triggered by an HT from a non-drosophilid species. However, an analysis of 99 long-read assemblies of different insect species did not identify any insertions similar to Blood, Opus, and 412 (*SI Appendix*, Fig. S16 and Table S5). To gain further insights into the direction of the HT, we performed a phylogenetic analysis with the TE insertions in *D. melanogaster* and species of the *D. simulans* complex. An HT from a species of the *D. simulans* complex to *D. melanogaster* should have led to a distinct topology of the phylogenetic tree, where *D. melanogaster* insertion is nested within insertions from the *D. simulans* complex. We extracted full-length insertions from long-read assemblies of two *D. melanogaster* strains, two *D. simulans* strains, one *Drosophila mauritiana*, and one *Drosophila sechellia* strain. We aligned the sequences of the insertions with Muscle and generated trees with BEAST (52, 53). First, we tested whether our approach can roughly reproduce the tree of the I-element in these species (20) (based on different assemblies and full-length as well as fragmented insertions). Similar to previous work, we found evidence for two waves of I-element invasions in *D. melanogaster*, a recent wave with short branches and an older wave with longer branches (*SI Appendix*, Fig. S17). Insertions from both waves are embedded within insertions from species of the *D. simulans* complex [*SI Appendix*, Fig. S17; (20)]. Furthermore, insertions of *D. simulans* and *D. sechellia* are frequently interleaved, again as shown before (20). Next, we investigated the trees for Blood, 412, and Opus (*SI Appendix*, Fig. S17). Based on the short branch lengths, we found evidence for recent waves of invasions for Opus, Blood, and 412 in *D. melanogaster*. Interestingly, 412 additionally has many insertions with short branches in *D. simulans*, suggesting a recent 412 invasion in *D. simulans* (*SI Appendix*, Fig. S17). For all three TEs, insertions with short branches in *D. melanogaster* were largely nested within insertions with longer branches from a species of *D. simulans* complex, consistent with an HT from a species of *D. simulans* complex to *D. melanogaster* (*SI Appendix*, Fig. S17).

In summary, we argue that a species from the *D. simulans* complex is the most likely source of the HT that triggered the invasions of Blood, Opus, and 412 in natural populations of *D. melanogaster*.

## Discussion

Taking advantage of publicly available genomes from historical *D. melanogaster* specimens, we showed that the LTR retrotransposons Blood, Opus, and 412 invaded natural *D. melanogaster* populations between ≈1850 and 1933. Solely a few degraded reads aligned to these TEs in any specimen collected until ≈1850, but a substantial number of reads with a high similarity to the consensus sequence aligned to these TEs in all specimens collected in 1933 (Fig. 1). This finding is robust to different methods for estimating TE copy numbers and to different read length (*SI Appendix*, Figs. S6 and S7). The presence of multiple full-length insertions with a high similarity to the consensus sequence in different long-read assemblies of different *D. melanogaster* strains also supports the view that Blood, Opus, and 412 were recently active (*SI Appendix*, Fig. S8). A high similarity among insertions of Blood, Opus, and 412 was also noticed in previous works (Opus=Nomad) (24). Insertions of Blood, Opus, and 412 are largely segregating at a low frequency in natural *D. melanogaster* populations, which further suggests that these TEs were recently active (54, 55). Based on samples collected in Europe, we estimate that Blood, Opus, and 412 invaded *D. melanogaster* between 1850 and 1933. However, the timing of these invasions might differ among geographic regions. In future work, it will be important to sample *D. melanogaster* genomes more densely in time and space to infer the invasion history of these TEs in different geographic regions.

By analyzing strains and specimens collected at different times, we provide an updated history of TE invasions *D. melanogaster* populations during the last 200y. Our data suggest that seven TEs invaded natural *D. melanogaster* populations during the last 200y. The four oldest invasions—Blood, Opus, 412, and Tirant which spread between 1850 and 1950—were due to LTR retrotransposons (Fig. 2). Our findings thus provide strong support for previous work suggesting that most LTR retrotransposons in *D. melanogaster* are of very recent origin [<100,000y, possibly even <16,000y (24)]. We suggest that the invasions of Blood, Opus, and 412 were triggered by HT from a different species. An alternative explanation could be that these TEs were already present at low numbers in *D. melanogaster* populations and were recently reactivated. This scenario can be excluded for Opus and 412 since we suggest that no full-length insertions of these TEs were present around 1800. For Blood, where we found some old full-length insertions, the phylogenetic analysis shows that the consensus insertions are more closely related to insertions from the *D. simulans* complex than to the old insertions, making HT again the more likely explanation.

We found that species from the *D. simulans* complex carry insertions that are very similar to the consensus sequences of Blood, Opus, and 412 in *D. melanogaster* (Fig. 4) and that recent insertions of *D. melanogaster* (short branches) are nested within older insertions (long branches) from species of the *D. simulans* complex (*SI Appendix*, Fig. S17). Therefore, we propose that HT from a species of the *D. simulans* complex triggered the invasion of Blood, Opus, and 412 in *D. melanogaster*. Given that the invasions of Blood, Opus, and 412 happened around the same time (1850 to 1933), we wondered whether these three invasions could have been triggered by a single event, such as an introgression from *D. simulans* into *D. melanogaster*. Our finding that the three TEs have insertions with high similarity in species of the *D. simulans* complex is consistent with a common origin of the three invasions. Further genomes of historical specimens collected between 1850 and 1933 at different geographic locations will increase the resolution for this critical period and might thus help to resolve this issue.

We suggest that 6 out of 7 TEs which invaded *D. melanogaster* during the last 200y were likely triggered by HT from a species of the *D. simulans* complex (Blood, Opus, 412, Tirant, I-element, and Hobo). A possible explanation for the high number of TE invasions triggered by HT from a species of the *D. simulans* complex is that TEs may be frequently horizontally transmitted back-and-forth between related species (20). As a consequence, TEs from related species may periodically reinfect each other, thus ensuring the long-term persistence of the TEs (20). The presence of degraded insertions in addition to full-length insertions for these six TEs (*SI Appendix*, Fig. S8) suggests that these TEs invaded *D. melanogaster* in multiple waves, in agreement with this hypothesis (20).

Although HT is occurring frequently among insect species (21), our work raises the important open question of whether seven TE invasions in 200y (one invasion all ≈30y) are representative of the evolution of *D. melanogaster*. The observed 200y could represent an unusual accumulation of TE invasions. If we roughly interpolate the rate of invasions (one TE invasion all 30y), the ≈121 TE families found in the genome of *D. melanogaster* could have been acquired by HT during the last 3,600y. However, given that we can still identify TEs that invaded the *D. melanogaster* genome 2 to 5 mya, such as Ine1, Jockey2, Helena, or Cr1a (24, 56), we would expect one invasion all 16,000y (2 million years/121 families) or one invasion all 8,000y if two waves of invasions are assumed for each TE family. We thus think that the rate of TE invasions observed during the last 200y in *D. melanogaster* is unusually high. This raises the question of which events might have triggered such a high rate of HTs in the last 200y. One possible explanation could be the recent habitat expansion of *D. melanogaster* into the Americas and Australia (26, 57). *D. melanogaster* originated in tropical sub-Saharan regions of Africa, started to colonize the rest of the World about 10,000 y ago, spread from the Middle East into Europe about 1,800 y ago, and finally spread to the Americas and Australia about 100 to 200 y ago (58–61). Habitat expansion may bring species into contact that were previously isolated, thus generating novel opportunities for HT among species. An illustrative example is the P-element in *D. melanogaster*, which was likely acquired from *D. willistoni* after *D. melanogaster* entered the habitat of *D. willistoni* in South America (62). Additionally, habitat expansion will increase the population size of species, and it may bring species into contact with novel vectors of HT, thereby increasing the opportunities for HT among species. The fact that Blood, Opus, and 412 invaded around the same time as *D. melanogaster* spread into North America and Australia argues in favor of the habitat expansion. A related question is whether the observed high rate of TE invasions during the last 200y can also be found in other species. For example, the habitat expansion of many species caused by human activity could have greatly accelerated the rate of TE invasions due to novel opportunities for HT, in both the species that expanded its habitat and the species whose ancestral habitat has been invaded. It will be important to test this hypothesis with additional species. Although strains sampled at different time points will only be available for few species, an analysis of historical museum specimens could in principle be feasible for many diverse species (63).

Out of the four TEs that invaded *D. melanogaster* during the last century, three TEs—the I-element (non-LTR), Hobo (DNA transposon), and the P-element (DNA transposon)—cause diverse hybrid dysgenesis (HD) effects. Crosses among males having the TE with females not having the TE typically lead to offspring where the TE is active and this TE activity can lead to different phenotypic effects such as atrophied ovaries (27, 64). This raises the question of whether Blood, Opus, and 412 could also induce HD symptoms. Answering this question requires both, strains that have these TEs and strains that do not have them. Since the oldest available lines of *D. melanogaster* were collected around 1925 to 1933, we do not have any strains that are devoid of recent Blood, Opus, and 412 insertions. The question could thus solely be answered by artificially introducing these TEs into naive strains [for example, using a different species such as *Drosophila erecta* (19)]. Given that we did not detect any HD symptoms for Tirant, i.e., the sole LTR retrotransposon that invaded during the last 100y (26), we suspect that the LTR retrotransposons Blood, Opus, and 412 might also not induce any HD symptoms.

Based on SNPs found in the TEs, we show that the composition of Opus and 412—but not of Blood—varies among populations. For Opus, specimens from Zimbabwe and Tasmania form distinct clusters and for 412 specimens from Zimbabwe and Beijing (Fig. 3). A previous work based on PCAs additionally found that the composition of Tirant, but not of the I-element, Hobo, and the P-element, varies among populations (26). Here, we confirm that the composition of Tirant varies among populations and that the I-element, Hobo, and the P-element do not show population structure (*SI Appendix*, Fig. S18). We think that two different processes could lead to a heterogeneous TE composition among extant populations, founder effects during the invasion, and demographic processes. An analysis of neutral autosomal SNPs revealed that the populations from Zimbabwe and Beijing form distinct groups [based on the first two principal components (42)], which is very similar to the population structure that we observed for 412 (and to a minor extent for Opus, where solely the population from Zimbabwe forms a distinct cluster). This raises the possibility that demographic processes shaped the composition of TEs in the extant population. There are however two problems with this hypothesis. First, the geographic pattern varies among the TE families (e.g., Tasmania is a separate cluster for Tirant, while Zimbabwe is a distinct cluster for Opus and 412), and several TE families show no discernible geographic pattern (Blood, I-element, Hobo, and P-element). If demographic processes shaped the TE composition, we expect that all TE families show the same geographic pattern. Second, Opus and 412 invaded *D. melanogaster* populations during 1850 to 1933. If demographic processes shaped the TE composition, then they must have accomplished this during the last 150y, which then raises the possibility that the geographic pattern seen with autosomal SNPs was also generated during these last 150y. However, the pattern seen for neutral autosomal SNPs is likely due to the out-of-Africa migration of *D. melanogaster* several thousand years ago (42) and not the result of recent demographic processes. We thus favor the hypothesis that founder effects during the invasions of the TEs are responsible for the observed geographic pattern seen for Tirant, Opus, and 412. For example, a few Opus insertions with a slightly different composition than the majority of the Opus insertions may have triggered the invasion of populations from Zimbabwe. As a result, the populations from Zimbabwe will end up with a slightly different TE composition than other populations (similar to a founder effect when a new population is established). The geographic pattern observed for 412 (and to some extent for Opus) and the neutral autosomal SNPs might thus have emerged twice independently. The similarity of the pattern could just reflect the fact that invading TEs and migrating flies need to overcome the same barriers (e.g., the Sahara).

The seven TE invasions during the last 200y had a substantial impact on the *D. melanogaster* genome. Due to these invasions, the genome size of *D. melanogaster* increased by up to 1.2 Mb in a short period of time (*SI Appendix*, Table S4). These novel TE insertions could provide variation driving adaptation (65, 66), generate novel piRNA clusters [especially Blood and 412 frequently form “de novo” clusters (67)], remodel gene regulatory networks (68), and generate diverse structural variants (69). The high rate of TE invasions during the last 200y may thus have had a substantial impact on the evolution of *D. melanogaster*.

## Materials and Methods

### Analysis of Genomic DNA.

We analyzed the TE content in genomic DNA of *D. melanogaster* samples from three different publicly available datasets: the Global Diversity Lines (42) (accession number: PRJNA268111), lab strains collected at different times (26) (accession number: PRJNA634847), and the historical museum specimens (36) (accession number: PRJNA945389). For an overview of the analyzed samples, see *SI Appendix*, Tables S1 and S2. We downloaded the files using wget, checked the md5 sum, and trimmed the reads to 100bp. To investigate the robustness of our results, we performed an additional analysis where reads were trimmed to 50bp. The reads were mapped to a database consisting of the consensus sequences of TEs (37) and three single-copy genes (*rhino*, *trafficjam*, and *rpl32*) with bwa bwasw (version 0.7.17-r1188) (70). Several of these analyses were parallelized with GNU parallel (71). We used DeviaTE (v0.3.8) (38) to estimate the copy numbers of TEs and to visualize the abundance and the diversity of TEs. DeviaTE estimates the copy numbers of TEs in haploid genomes by normalizing the coverage of a TE sequence to the coverage of single-copy genes. To estimate the number of reads mapping to each TE (reads per million mapped reads; rpm), we used PopoolationTE2 v1.10.03 (72).

To identify TE insertions in the high-quality assemblies of the *D. melanogaster* strains [Canton-S, Iso1, Pi2, and Dgrp-732 (39–41)], we used RepeatMasker [open-4.0.7; -no-is -s -nolow; (73)] providing the consensus sequences of TEs (37) as custom library. We merged fragmented matches using a Python script (rm-defragmenter.py–dist 100) and visualized the joint distribution of the insert size and the divergence using hexagonal heatmaps [ggplot2 (74)].

### PCA.

In order to identify population structure in the GDL samples, we estimated the frequencies of TE-specific SNPs, which were inferred from reads aligned to the consensus sequences of TEs (see above). This frequency will reflect the TE composition in a given sample. For example, if a specimen has 10 Opus insertions and a biallelic SNP with a frequency of 0.6 in Opus at position 351, then about 6 Opus insertions in the sample will have the SNP and 4 will not have it. We estimated the allele frequency of TE-specific SNPs in the GDL samples with DeviaTE (38). We filtered the SNPs by solely using biallelic SNPs and removing SNPs solely found in few samples (≤3 samples) using a Python script (mpileup2PCA.py). These filtered SNPs were then subjected to multidimensional analysis in R, using PCA (prcomp).

### piRNAs.

We utilized data from 10 GDL strains (43) for the piRNA analysis. We removed the adaptor sequence “TGGAATTCTCGGGTGCCAAGG” using cutadapt [v4.4 (75)] and filtered for reads having a length between 18 and 36 nt. Subsequently, the reads were aligned to a database encompassing *D. melanogaster* miRNAs, mRNAs, rRNAs, snRNAs, snoRNAs, tRNAs (76), and TE sequences (37) using novoalign (v3.09.04). To compute the ping-pong signatures and visualize the piRNA abundance along the sequence of the TEs, we employed previously developed Python scripts (19).

### Origin of the HTs.

To identify potential donor species for the HT of Blood, Opus, 412, Tirant, the I-element, Hobo, and the P-element, we investigated the long-read assemblies of 101 diverse drosophilid species (45) and of 99 different insect species (77) (*SI Appendix*, Table S5). We included the long-read assemblies of a recently collected *D. melanogaster* (Pi2) and *D. simulans* (SZ232) strain into the analysis (46, 47). The assemblies were downloaded with NCBI datasets (v14.24.0). We used RepeatMasker (73) (open-4.0.7; -no-is -s -nolow) with the consensus sequences of TEs (37) as custom library to identify TE insertions in these assemblies. A Python script was used to identify for each assembly and for each TE family the best match (i.e., the HSP with the highest alignment score) (process-101genomes.py). The script further computes for each TE family the similarity of the best match to the consensus sequence as $s=max\left(rms_{i}\right)/max\left(rms_{\mathrm{all}}\right)$, where $max(rms_{i})$ is the highest RepeatMasker score (rms) in a given assembly (i) and $max(rms_{\mathrm{all}})$ the highest score in any of the assemblies. The similarity is a value between 0 and 1, where 0 indicates no similarity to the consensus sequence of the TE and 1 a high similarity.

### Phylogenetic Trees.

To generate phylogenetic trees for the I-element, 412, Blood, and Opus, we used RepeatMasker (open-4.0.7; -no-is -s -nolow) to identify insertions of these TEs in long-read assemblies of the 101 drosophilid species (45). We extracted the sequences of mostly full-length insertions (based on a length threshold; for Blood and 412: 6,000 to 8,000bp; for Opus: 5,000 to 8,000bp; for the I-element: 4,000 to 6,000) with bedtools (78) (v2.30.0) and performed multiple sequence alignment using MUSCLE (v3.8.1551) (52). The tree was generated with BEAST (v2.7.5) (53).

## Supplementary Material

Appendix 01 (PDF)

## Data Availability

All analyses performed in this work were documented in RMarkdown and have been made publicly available, together with the resulting figures, at GitHub (https://github.com/Almo96/dmel_TE_invasions) (79) (see *.md files). All the data used are previously mentioned and a careful description of the strains is available in the supplementary tables.
