# Supplementary material for: Genomes of historical specimens reveal multiple invasions of LTR retrotransposons in Drosophila melanogaster during the 19th century
Source: Proc Natl Acad Sci U S A. 2024 Apr 2;121(15):e2313866121. doi: 10.1073/pnas.2313866121 (PMC11009621; doi:10.1073/pnas.2313866121)
Supplement: Supplementary file 1 — Appendix 01 (PDF) [file pnas.2313866121.sapp.pdf]

Supplementary figures and tables

Almorò Scarpa, Riccardo Pianezza, Filip Wierzbicki and Robert Kofler

Supplementary figures

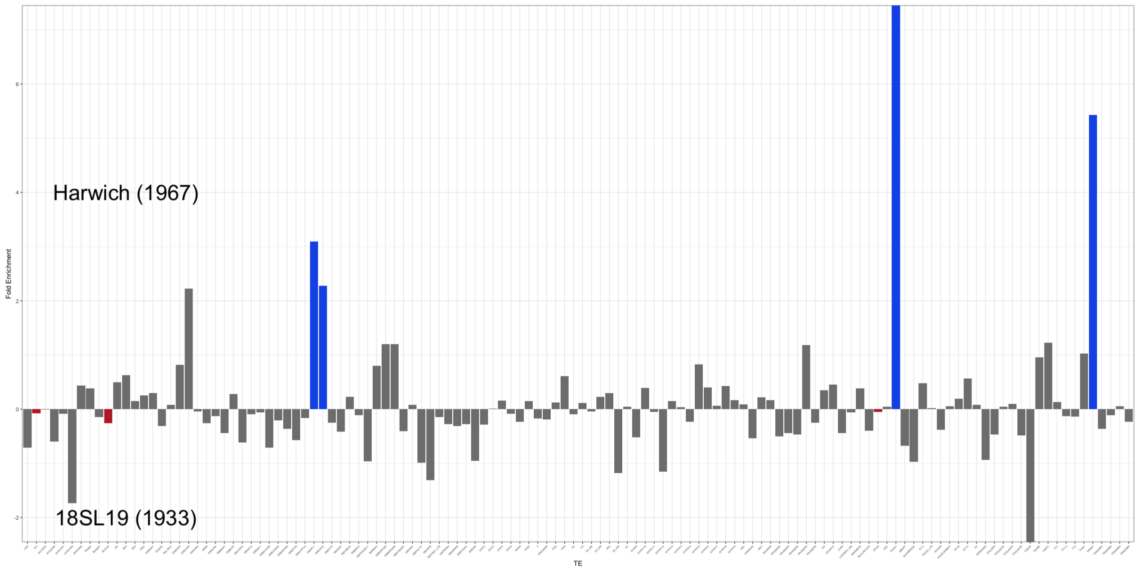

Figure 1: Differences in TE abundance between a strain collected in 1967 (Harwich) and 1933 (19SL19). For each TE family (x-axis) we show the difference in TE copy numbers per haploid genome as estimated with DeviaTE (y-axis). As expected Hobo, the I-element, the P-element and Tirant (blue bars) are overrepresented in Harwich [Schwarz et al., 2021].

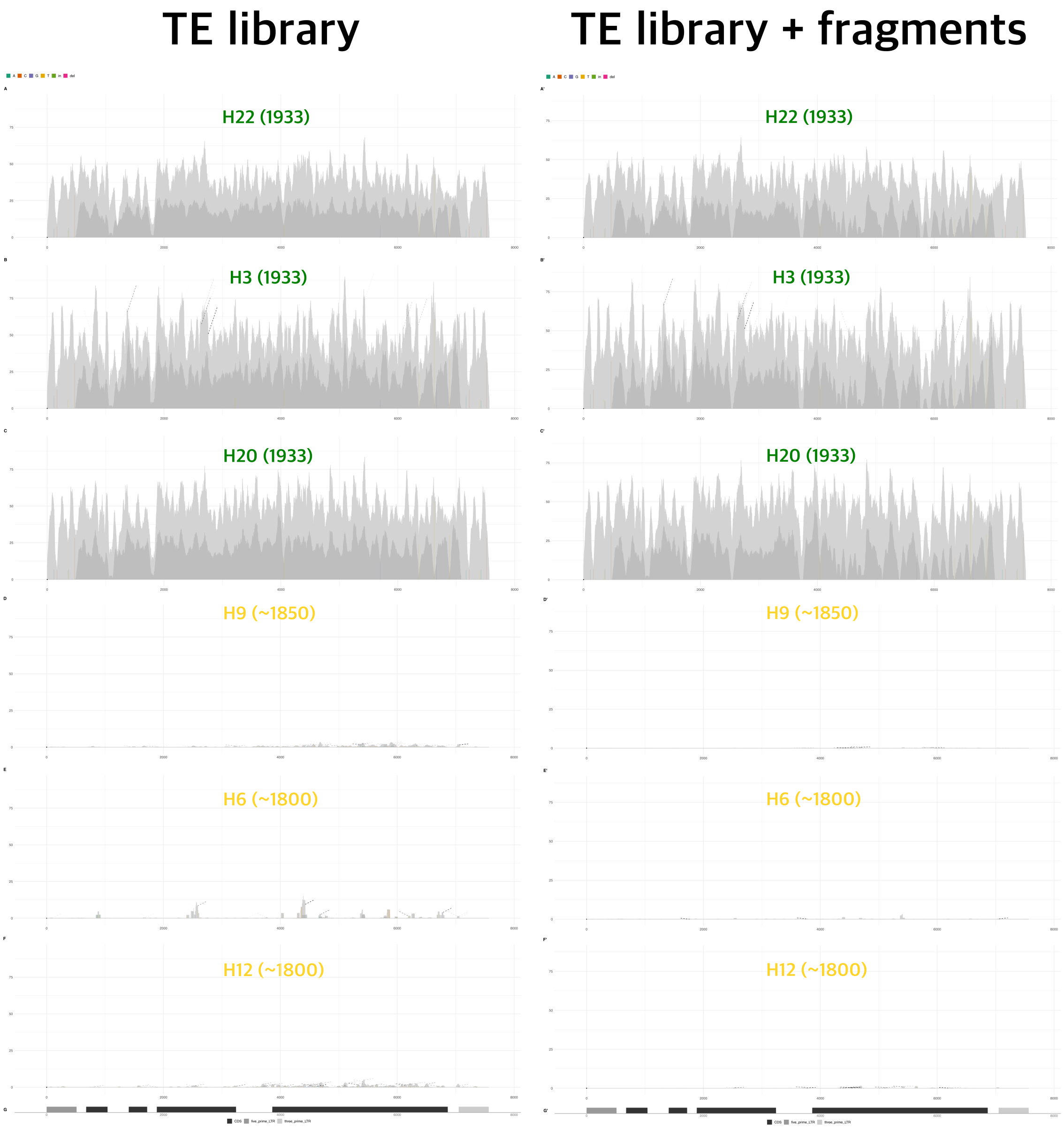

Figure 2: DeviaTE plots for 412 using three strains sampled around 1800 (top three) and three strains sampled around 1933 (bottom three). Plots are shown for an approach where just the consensus sequences of TEs are analysed (left panel) and an approach where the consensus sequences of TEs as well as the sequences of diverged TE fragments are analyzed (right panel). With both approaches, the DeviaTE plots are solely shown for the consensus sequence. The coverage of 412 was normalized to the coverage of single-copy genes. Single-nucleotide polymorphisms (SNPs) and small internal deletions (indels) are shown as colored lines. The coverage based on uniquely and ambiguously mapped reads is shown in dark and light gray, respectively. Note that solely few ambiguously mapped reads align to 412 in samples collected around 1800-1850 when the diverged fragments are included into the analysis.

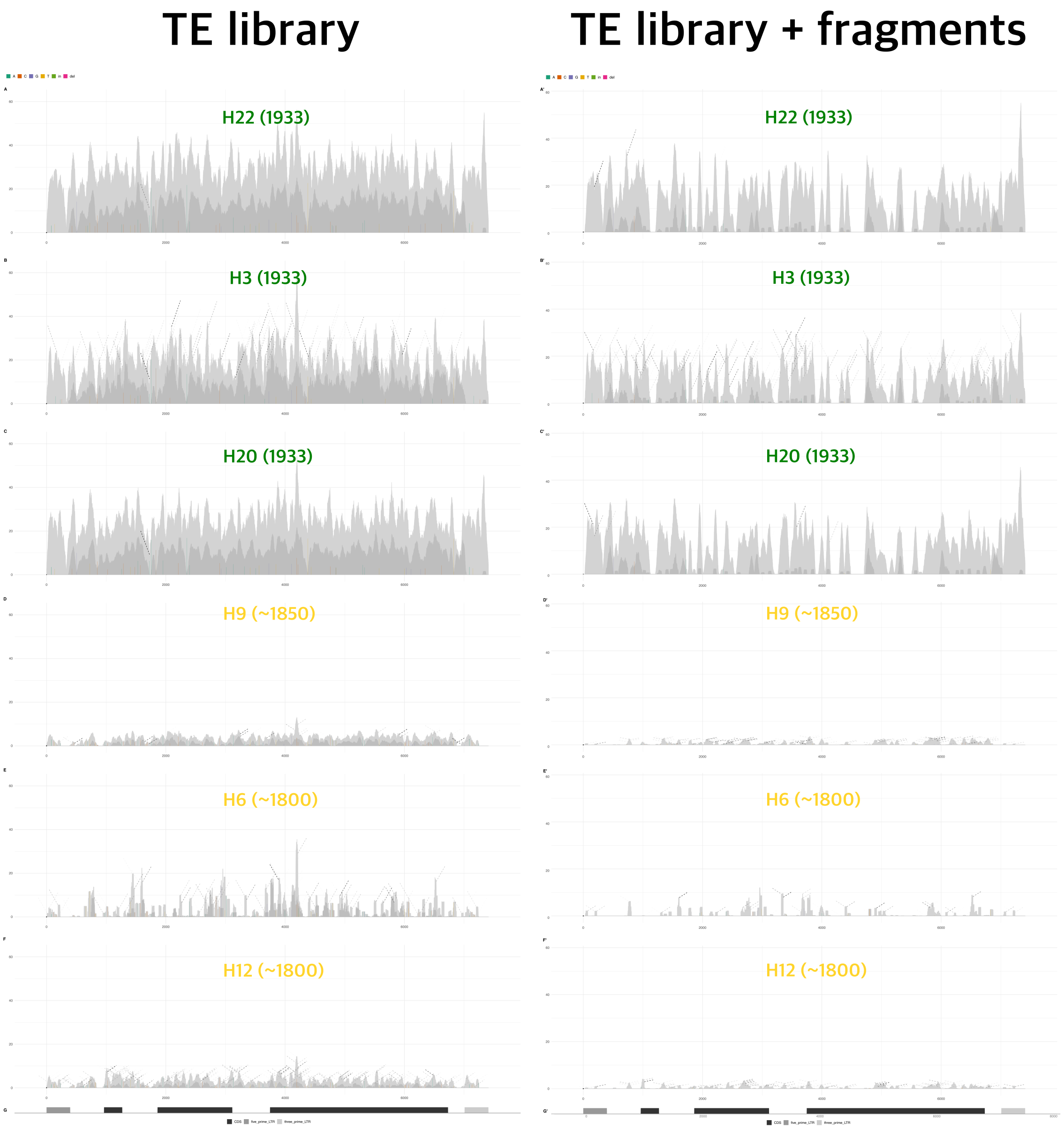

Figure 3: DeviaTE plots for Blood using three strains sampled around 1800 (top three) and three strains sampled around 1933 (bottom three). Plots are shown for an approach where just the consensus sequences of TEs are analysed (left panel) and an approach where the consensus sequences of TEs as well as the sequences of diverged TE fragments are analyzed (right panel). With both approaches, the DeviaTE plots are solely shown for the consensus sequence. The coverage of Blood was normalized to the coverage of single-copy genes. Single-nucleotide polymorphisms (SNPs) and small internal deletions (indels) are shown as colored lines. The coverage based on uniquely and ambiguously mapped reads is shown in dark and light gray, respectively. Note that solely few ambiguously mapped reads align to Blood in samples collected around 1800-1850 when the diverged fragments are included into the analysis. With this approach, reads unambiguously aligned to the consensus sequence of Blood are solely found in samples collected around 1933 (the coverage reduction is due to four 2%-diverged Blood insertions, which have a sequence that is highly similar to the consensus sequence and thus attract several reads during mapping).

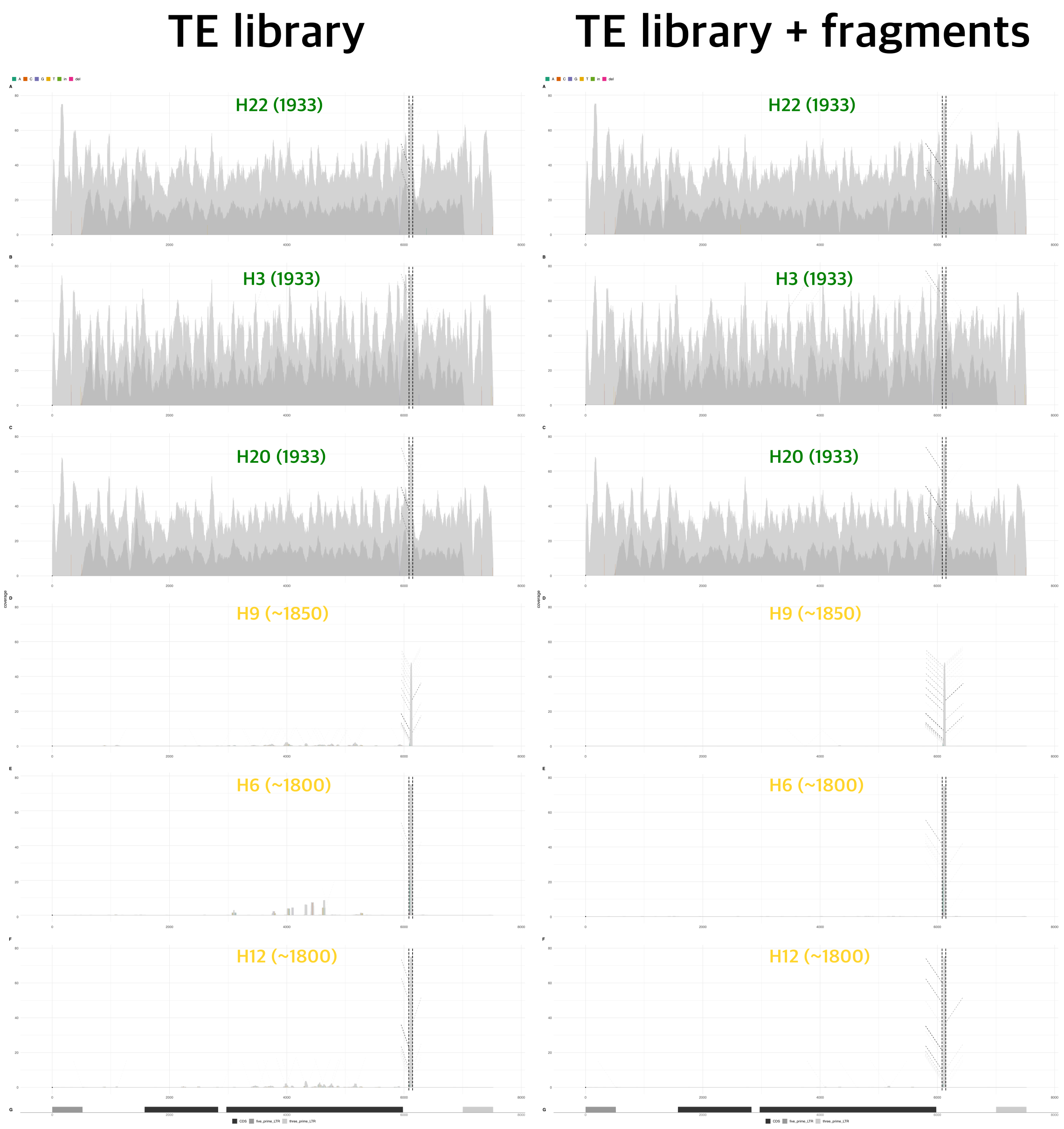

Figure 4: DeviaTE plots for Opus using three strains sampled around 1800 (top three) and three strains sampled around 1933 (bottom three). Plots are shown for an approach where just the consensus sequences of TEs are analysed (left panel) and an approach where the consensus sequences of TEs as well as the sequences of diverged TE fragments are analyzed (right panel). With both approaches, the DeviaTE plots are solely shown for the consensus sequence. The coverage of Opus was normalized to the coverage of single-copy genes. Single-nucleotide polymorphisms (SNPs) and small internal deletions (indels) are shown as colored lines. The coverage based on uniquely and ambiguously mapped reads is shown in dark and light gray, respectively. The coverage was manually curbed at the poly-A track (dashed lines). Note that almost no reads align to Opus in samples collected around 1800-1850 when the diverged fragments are included into the analysis.

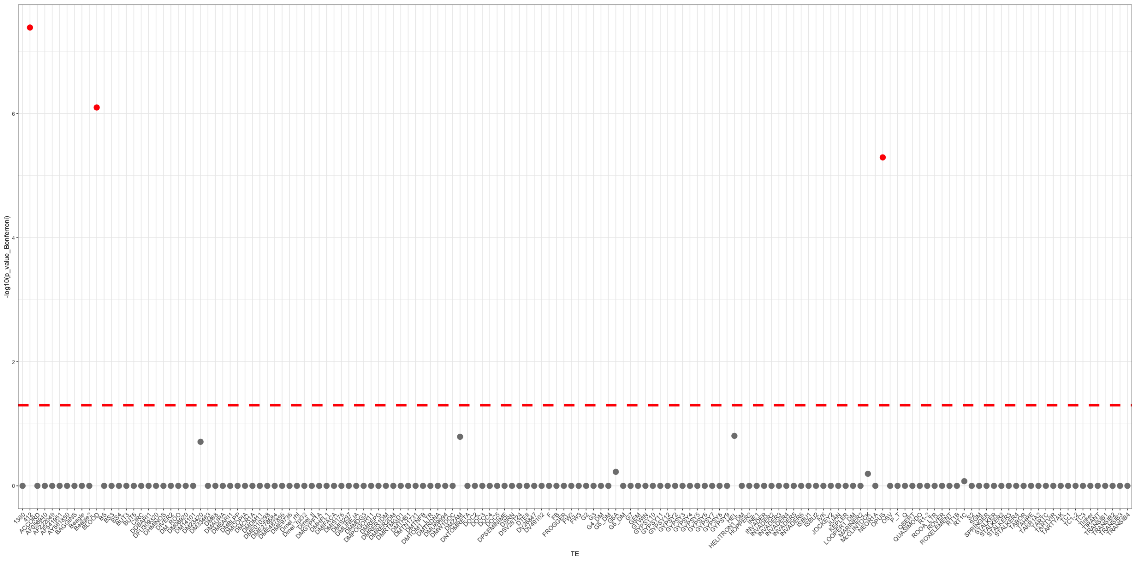

Figure 5: Significance of the copy number differences between samples collected in 1933 (16 females) and samples collected 1800 (6 females). Copy numbers were estimated with DeviaTE for all TE families and the significance was assessed with a t-test. After Bonferroni correction (red dashed line is the significance threshold, $P < 0.05$) solely Blood, Opus and 412 remain significant (red dots).

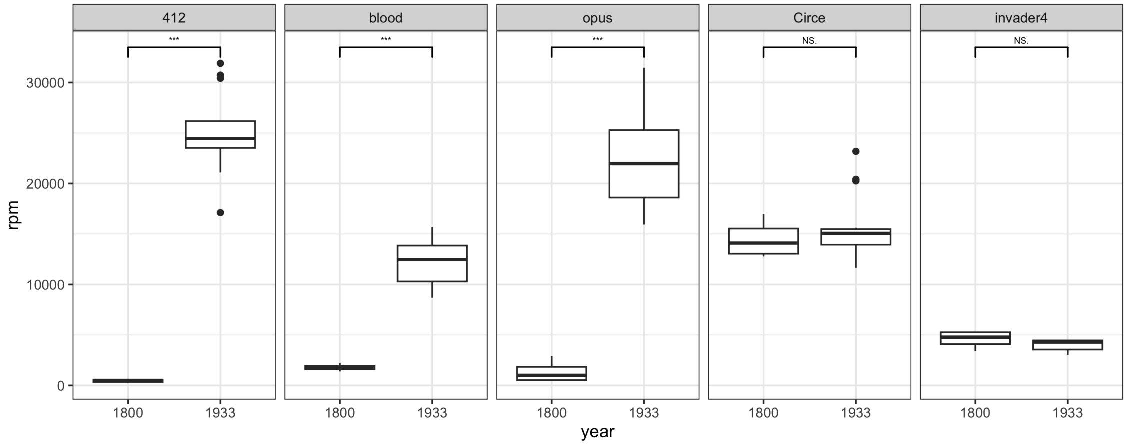

Figure 6: Abundance of 412, Opus and Blood in historical specimens collected around 1800 (6 samples) and 1933 (16 samples). As controls Circe and Invader-4 are included. The TE abundance is provided in reads mapping to the TE out of a million mapped reads (rpm). The significance was computed with Wilcoxon rank sum tests.

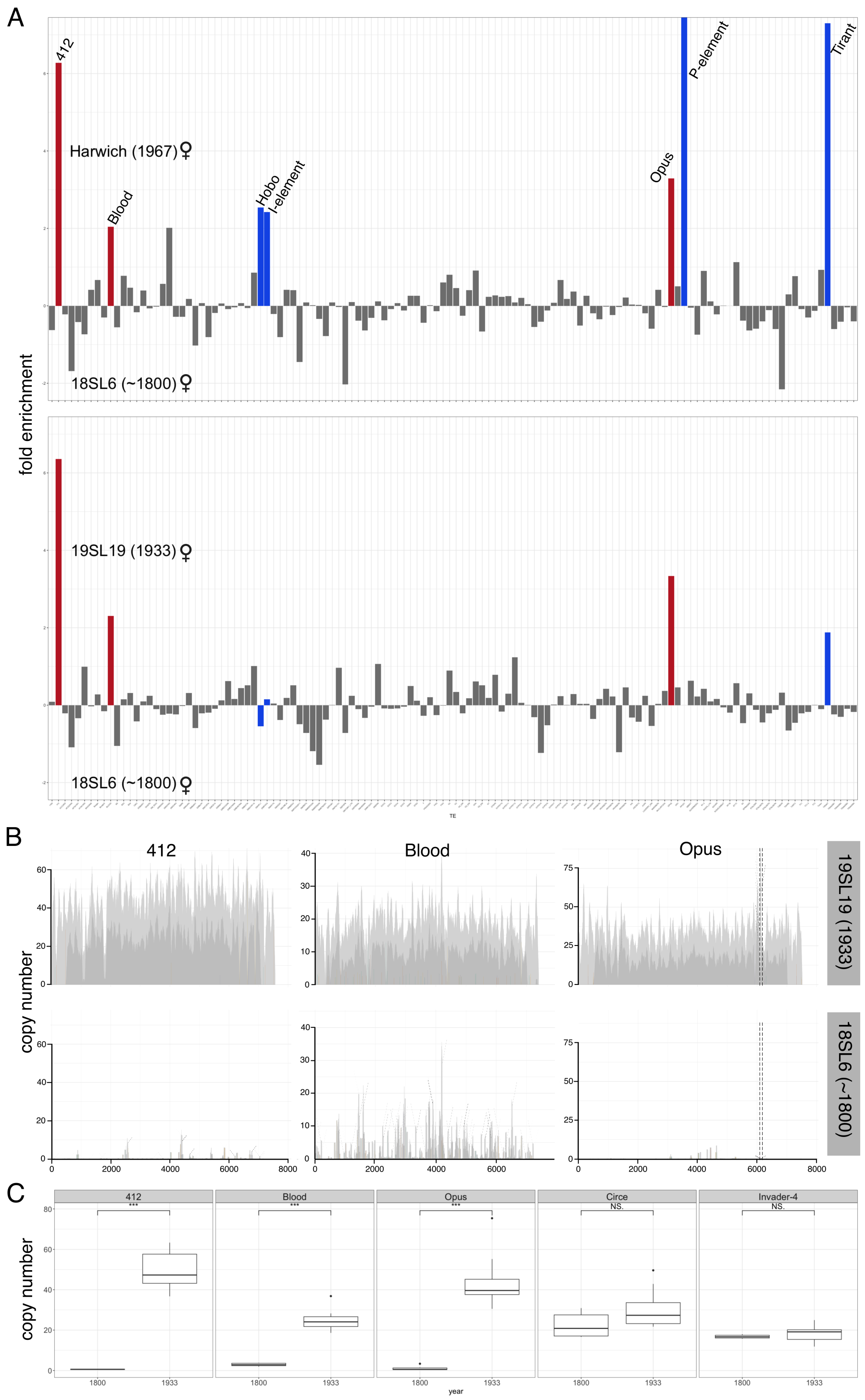

Figure 7: Reproducing figure 1 of the main manuscript with reads of length 50 (reads with a length of 100bp were used for the figure in the main manuscript). Note that the analysis of reads with 50bp length confirms that Blood, 412 and Opus have highly elevated copy numbers in strains sampled ≥ 1933 as compared to strains sampled around 1800.

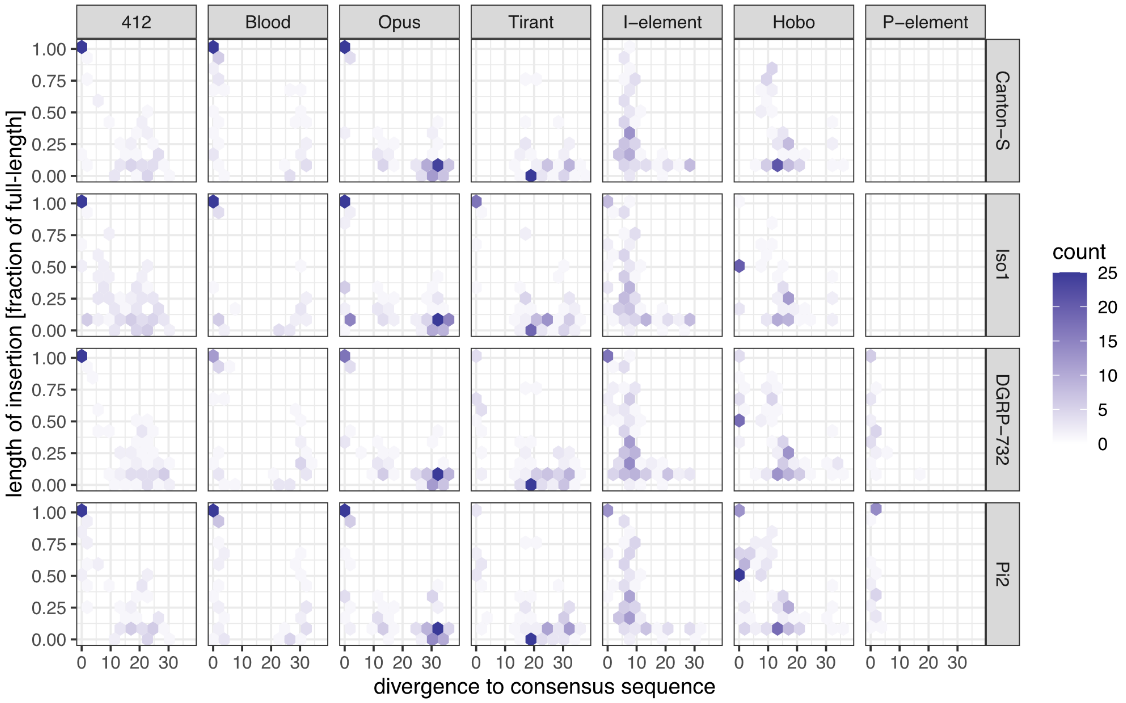

Figure 8: 2D-histograms showing the distribution of TE insertions in long-read assemblies of four different *D. melanogaster* strains. We plotted the length of the TE insertions (normalized to the full-length element) versus the divergence of the insertions to the consensus sequence. Recent insertions of a TE family are likely full-length and show little divergence (upper left corner), whereas older insertions are likely degraded and fragmented (lower right corner). Blood, Opus, and 412 may thus have recent insertions in all four analysed *D. melanogaster* strains. Note that Canton-S is an old lab strain and does therefore not have recent insertions of Tirant, I-element, Hobo and the P-element. As expected, Iso1 is lacking P-element insertions.

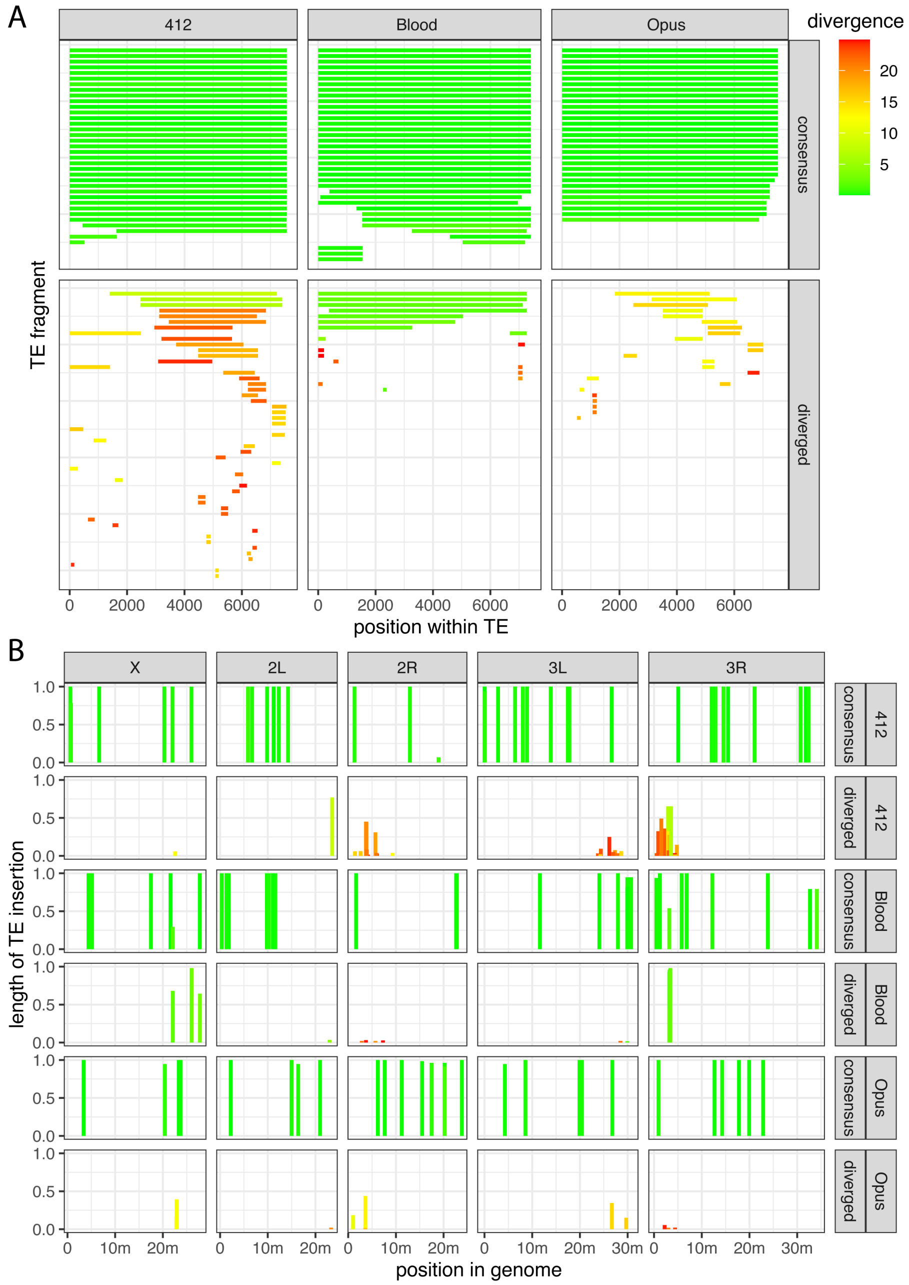

Figure 9: Overview of Blood, Opus and 412 insertions in a long-read assembly of Canton-S. A) All fragments matching with the consensus sequence of Blood, Opus and 412 (identified with RepeatMasker; divergence $< 25\%$). Horizontal bars represent the matching regions relative to the consensus sequence of the TE. Colors represent divergence from the consensus sequence. Based on a divergence threshold of 1.5% we classified the fragments into consensus ($\leq 1.5\%$) and diverged ($> 1.5\%$). B) Position of the TE fragments in the genome. Color coding and classification of insertions (i.e. consensus and diverged) are as described above. The length of the TE (normalized to the full-length element) is shown on the y-axis. Note that the diverged insertions of all three TEs are close to the ends of the chromosome arms (likely heterochromatic regions).

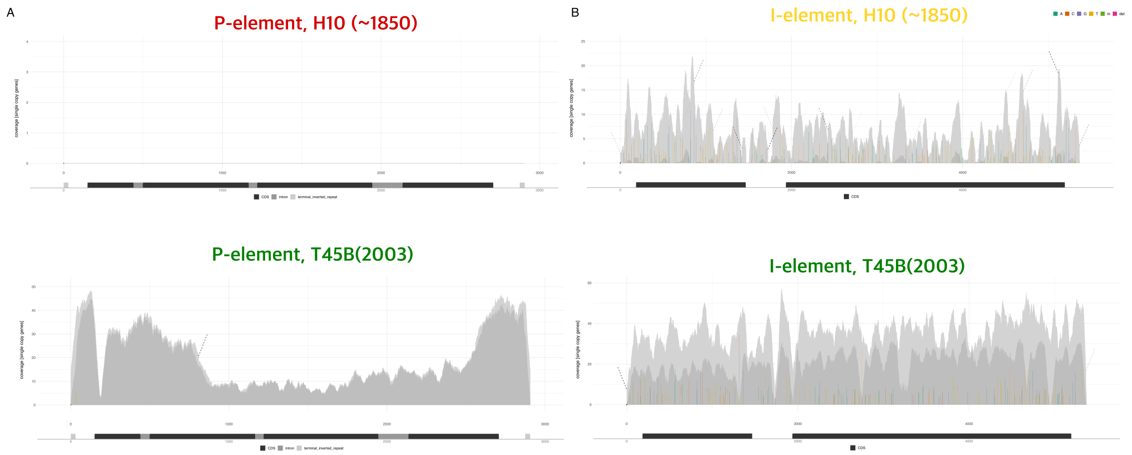

Figure 10: Examples for our classification of the status of TE invasions based on the well-established invasions of the P-element and the I-element [Kidwell, 1983]. Red: complete absence of any reads matching a TE; Yellow: only highly degraded reads align to a TE; Green: contiguous coverage of reads with a high similarity to the consensus sequence can be found (mostly in addition to the coverage generated by degraded fragments). These DeviaTE plots [Weilguny and Kofler, 2019] summarize the abundance and diversity of TEs in a sample (e.g. *D. melanogaster* strains collected at different times). To enable comparing samples with different sequencing depth the coverage of TEs was divided by the average coverage of single copy genes. As a result, the normalized coverage provides a proxy for the copy number of a TE. SNPs and indels are shown as colored lines. Coverage based on unambiguously and ambiguously aligned reads is shown in dark and light grey, respectively.

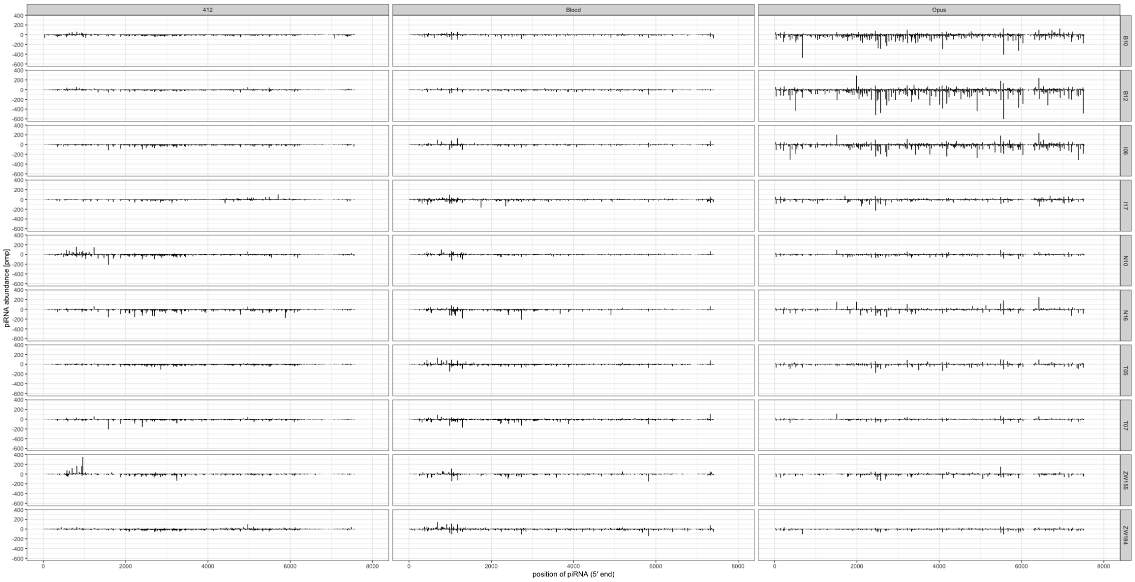

Figure 11: Distribution of piRNAs along Blood, Opus and 412 in 10 lines sampled from diverse geographic regions (GDL [Luo et al., 2020]). For each piRNA solely the 5' position is shown. Sense piRNAs are shown on the positive y-axis and antisense piRNAs on the negative y-axis. The total abundance of piRNAs was normalized to one million piRNAs.

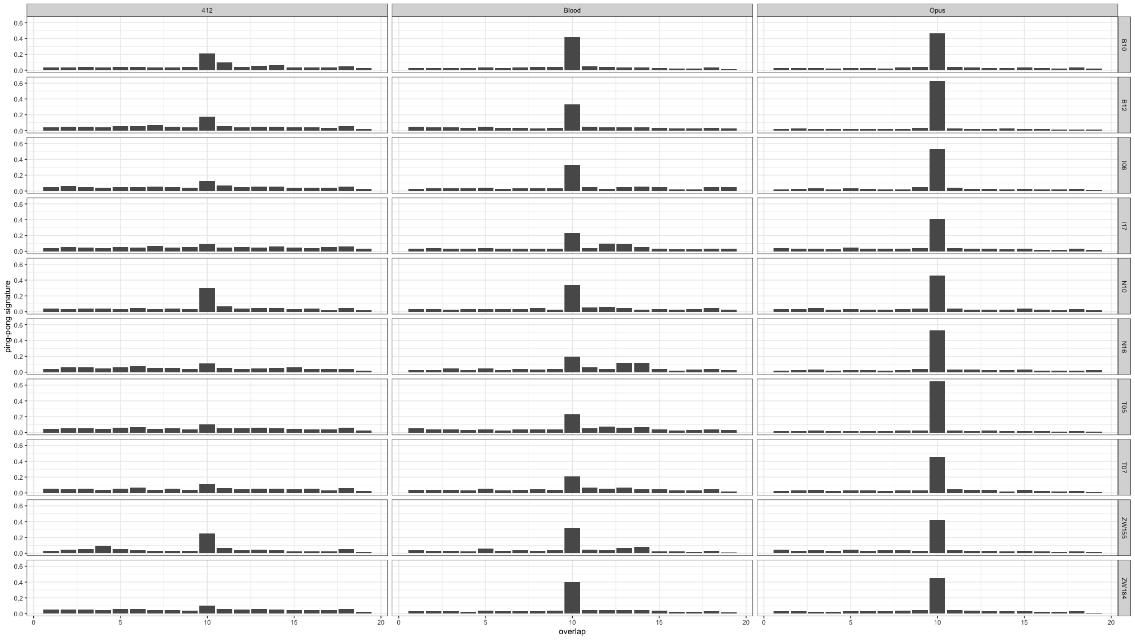

Figure 12: Ping-pong signatures for Blood, Opus and 412 in 10 lines sampled from diverse geographic regions (GDL [Luo et al., 2020]). These histograms show the distance between the 5' position of sense and antisense piRNAs. A peak at position 10 suggests that the ping-pong cycle is active and thus that the TE is silenced by the host defence.

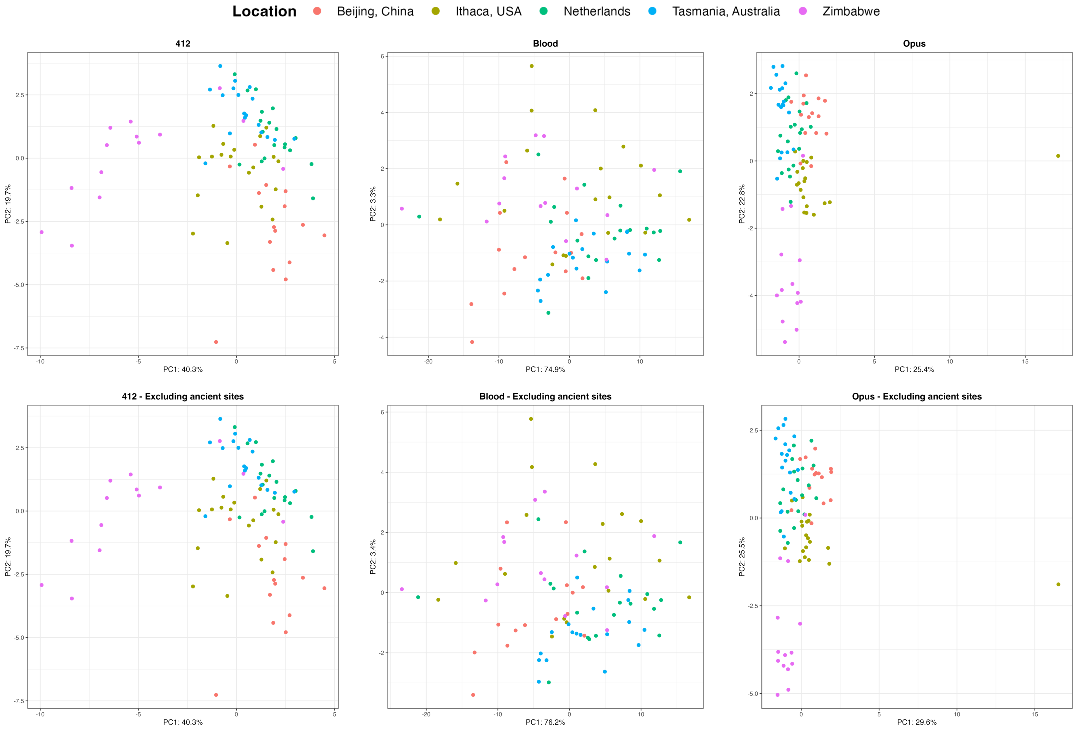

Figure 13: PCA for Blood, Opus and 412 in the 85 GDL strains including and excluding sites covered by ancient/degraded fragments of these TEs. We treated sites having a coverage in specimens collected around 1800 as ancient sites (due to the generally elevated coverage, we used a coverage threshold of > 5 for Blood). Note that excluding the ancient sites only had a minor impact on the overall pattern seen in the PCAs.

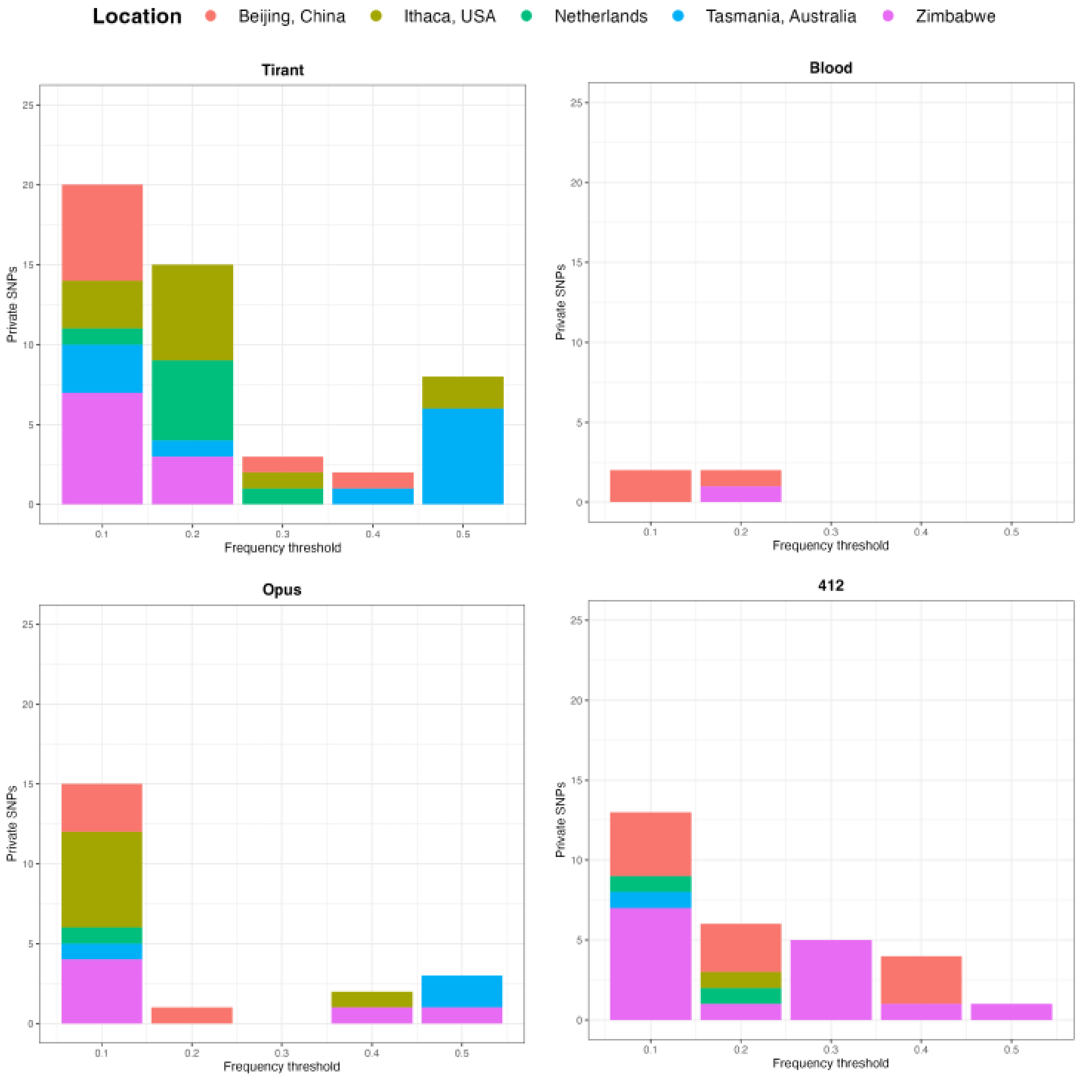

Figure 14: Frequency of diagnostic SNPs in the 85 GDL strains. We defined diagnostic SNPs to be abundant (≥50%) in a population of interest but rare in all other populations (<21%). The x-axis refers to the frequency of the diagnostic SNP in a GDL strain. For example with a frequency of 0.4 about 40% of the TEs in a strain carry the diagnostic SNP. Data are shown for Tirant, Blood, Opus and 412. Notably the abundance of the diagnostic SNP reflects the clusters observed in the PCAs (main manuscript).

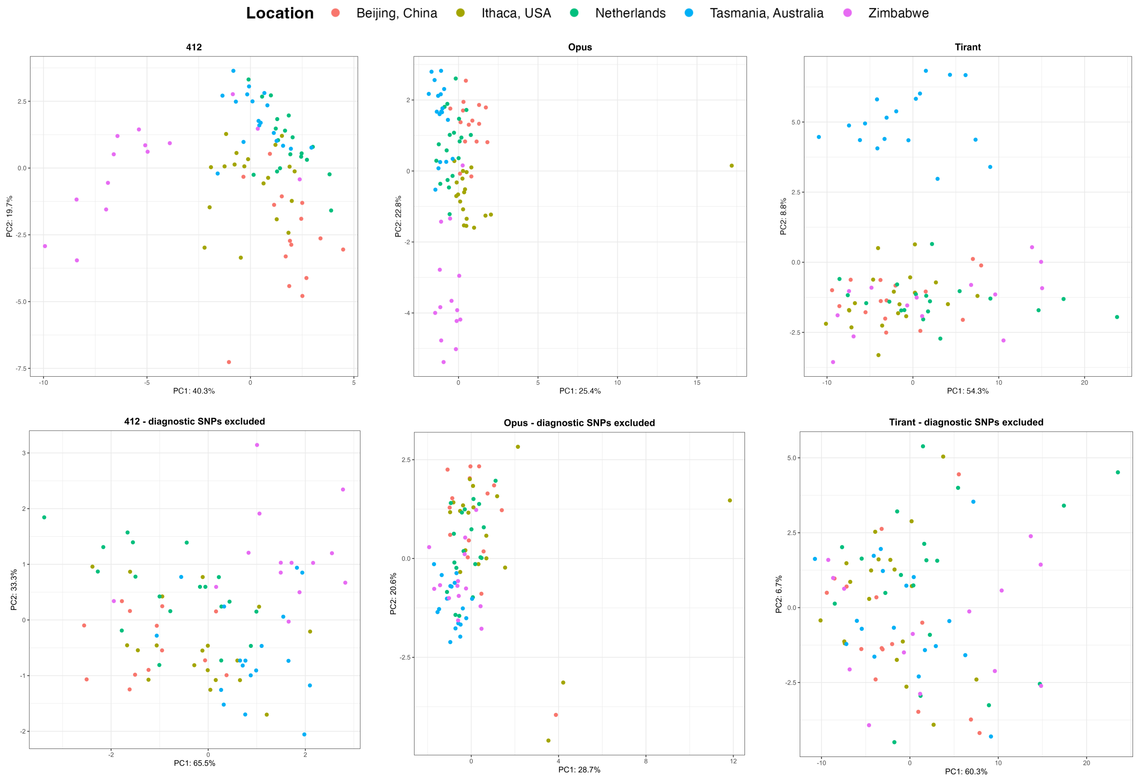

Figure 15: PCA for Blood, Opus and 412 in the 85 GDL strains including and excluding diagnostic SNPs. Note that excluding the diagnostic SNPs (defined as variants present in a population at a minimum frequency of 0.5 but only present at a maximum frequency of 0.2 in the others) led to a collapse of the geographic pattern seen in the PCAs.

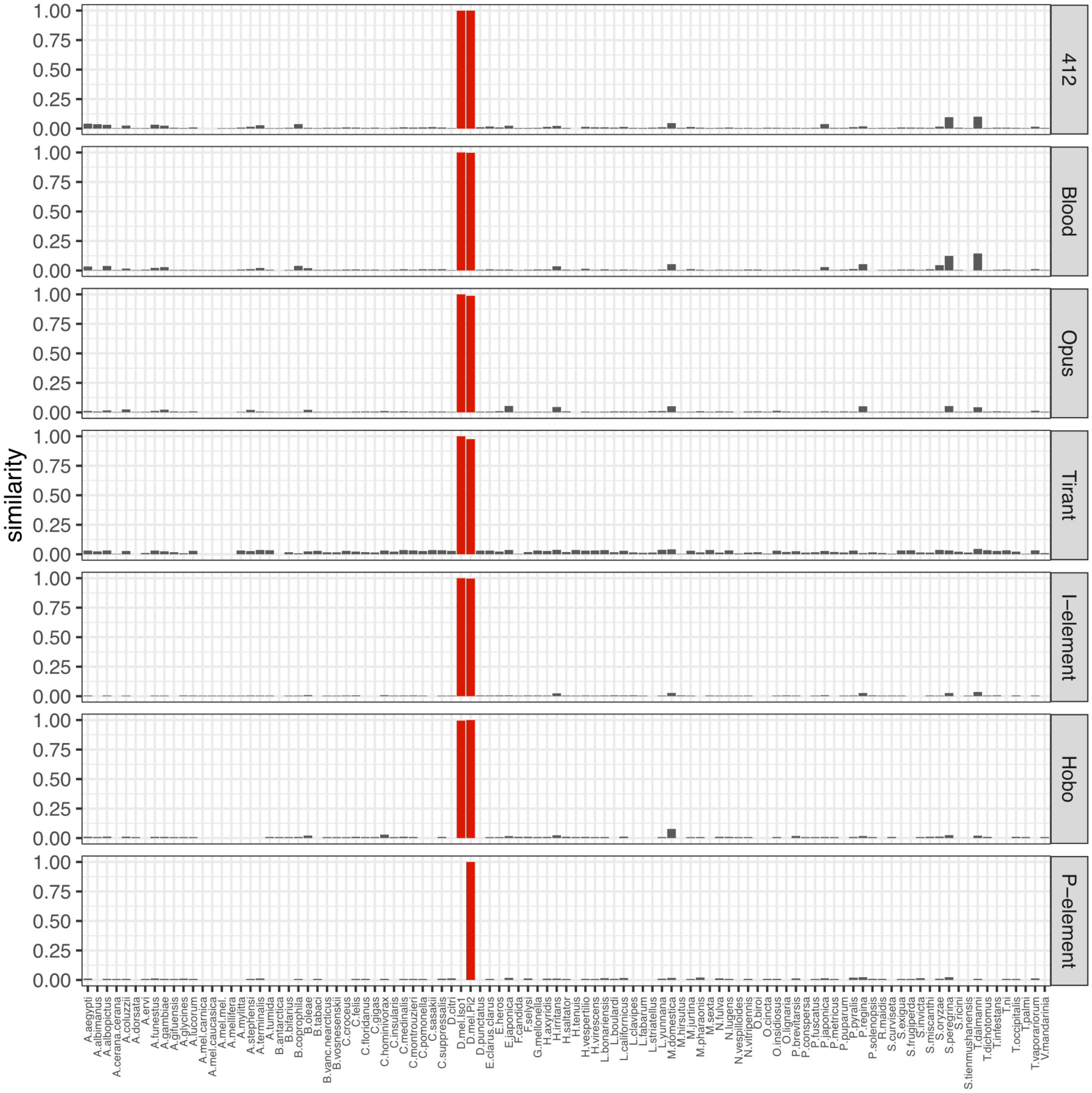

Figure 16: Barplots show the similarity of TE insertions in a given assembly to the consensus sequence of a TE for 99 long-read assemblies of diverse insect species. As reference, *D. melanogaster* (red) is included. For example, 0.9 means that at least one TE insertion in a given assembly has a high similarity ($\approx 90\%$) to the consensus sequence of the TE.

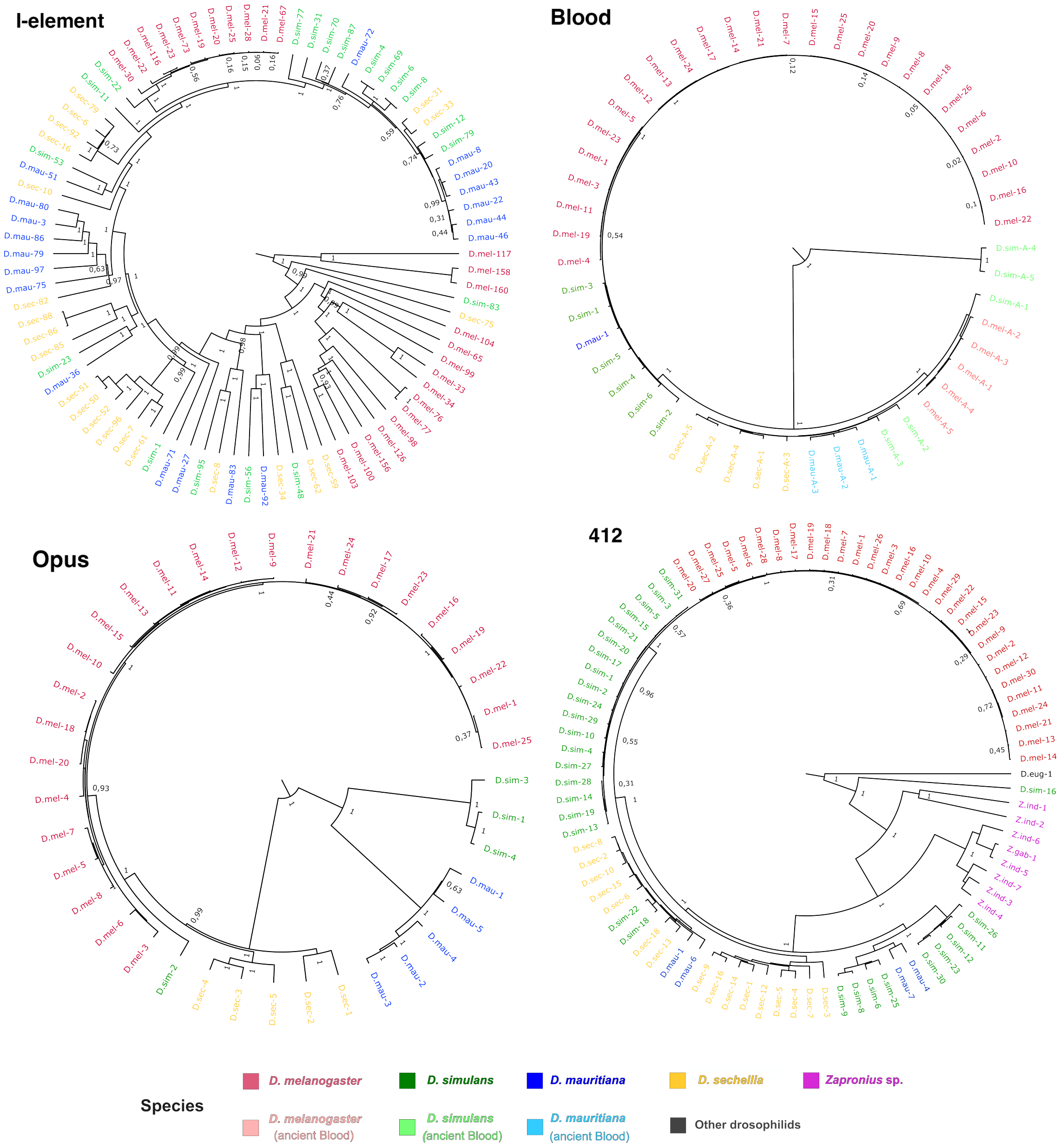

Figure 17: Phylogenetic trees for full-length insertions of the I-element, Blood, Opus and 412 in long read assemblies of 101 drosophilids. Note that for Blood, Opus and 412 short branches of *D. melanogaster* insertions are largely nested within longer branches of insertions in species from the *D. simulans* complex. This suggests that the HT occurred from a species of the *D. simulans* complex to *D. melanogaster*. Insertions of the ancient Blood (2%-diverged Blood) are forming a separate cluster with long branches. Ancient Blood insertion were identified using their specific LTR. Species of the genus *Zapronius* and *D. eugracilis* are also harbouring full length elements of 412, highly diverged from each other and from the *D. melanogaster* sequences. For each important node, the posterior probability is shown.

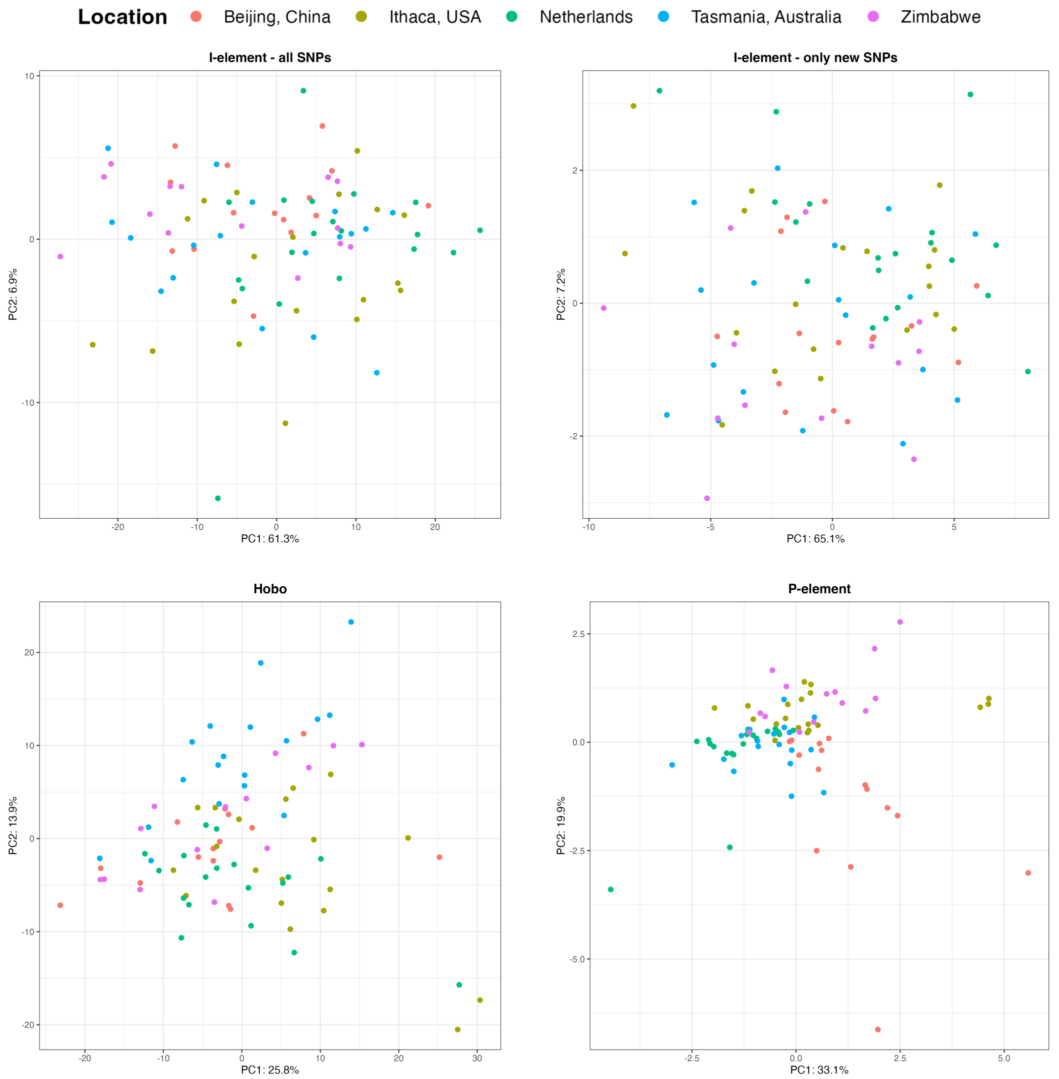

Figure 18: PCAs for the I-element, Hobo and the P-element in the 85 GDL strains. PCAs are based on the allele frequency of TE specific SNPs. The ancient I-element led to many diverse insertions which accumulated a substantial amount of SNPs. As these ancient SNPs could confound an analysis of the populations structure we performed PCA with and without the ancient SNPs for the I-element.

Supplementary results 1 - Blood

In historical samples collected around 1800 we found a continuous coverage along the sequence of Blood (supplementary fig. 3). This raises the question of what is responsible for this continuous coverage. We investigated the composition of Blood in the *D. melanogaster* strain Canton-S. Apart from the consensus insertions we found some highly diverged fragments (≈20% divergence) from parts of LTRs of the TE (supplementary fig 9). These highly diverged fragments only cover parts of Blood and thus cannot account for the continuous coverage seen in samples collected around 1800 (supplementary fig. 9). We noticed that Canton-S also has four full-length insertions of Blood with an average divergence of ≈ 2%. These 2%-diverged Blood insertions have slightly shorter LTRs (383bp vs 398bp) that are quite distinct from the LTRs of the consensus Blood (25% difference). Similar to the diverged fragments of Opus and 412, these 2%-diverged Blood insertions are close to the ends of the chromosome arms, i.e. likely heterochromatin (supplementary fig 9). We hypothesized that the 2%-diverged Blood insertions could already have been present in *D. melanogaster* by 1800 and thus account for the continuous Blood coverage observed in samples collected around 1800. To test this hypothesis we first estimated the age of the Blood insertions. Since, the 5' and the 3' LTR are synthesized from the same template during reverse transcription of LTR transposons, the sequence of both LTRs are identical when the LTR transposon integrates into the genome [Bowen and McDonald, 2001]. Therefore the age of LTR insertions can be easily estimated when the substitution rate (i.e. the rate of the molecular clock) is known, using the equation $T = K/2r$ where T is the age of the insertion K the divergence and r the substitution rate [Bowen and McDonald, 2001]. The 2% diverged Blood have $K = 0.01175$ (supplementary fig 19). Assuming $r = 0.0111$ mutations per site per million years [Tamura et al., 2004, Bergman and Bensasson, 2007] we estimate that the 2%-diverged Blood insertions are about 650.000 years old. By contrast the consensus insertions of Blood are much younger (27.000 years with $K = 0.000596$). Finally we tested if the 2%-diverged Blood insertions could account for the continuous coverage of Blood in samples collected around 1800. When we add the sequence of the four 2%-diverged Blood insertions (supplementary fig 9) to our pipeline, solely a few ambiguously mapped reads (50bp) align to the consensus sequence of Blood for samples collected around 1800 (supplementary fig 3). On the other hands, reads unambiguously aligned to the consensus sequence of Blood can only be found for samples collected around 1933. Therefore the 2% diverged Blood insertions could account for the continuous coverage of Blood seen in samples collected around 1800.

In summary, we found four old insertions of Blood (650.000 years) close to the heterochromatin that likely account for the continuous coverage of Blood in samples collected around 1800.

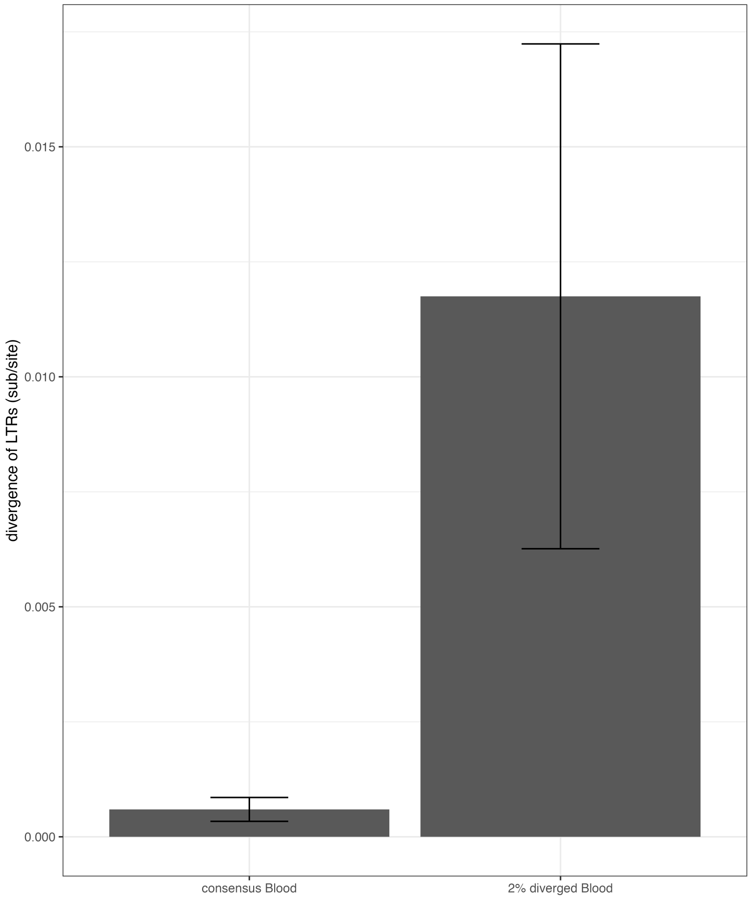

Figure 19: Age of the Blood insertions. Plots show the divergence between the 5' and the 3' LTR of Blood insertions. Data are shown for consensus insertions and 2%-diverged insertions. Based on the intra-element divergence between the 5' and the 3' LTR the age of the insertions can be estimated [Bowen and McDonald, 2001]. Based on these data we estimate that the 2%-diverged Blood insertions are about 650.000 years old, while consensus insertions of Blood are much younger.

Supplementary tables

Table 1: Overview of the TE composition in the strains used for reconstructing the history of TE invasions in *D. melanogaster* during the last 200 years. For each strain we show the SRA number, the approximate year and location of the collection and the abundance of the seven TEs that recently invaded *D. melanogaster* populations. The numbers refer to TE copy numbers per haploid genome (estimated with DeviaTE [Weilguny and Kofler, 2019]. We classified the TE content into three distinct categories: red absence of any TE sequence, yellow solely degraded TE sequences are present, green non-degraded TE sequences with a high similarity to the consensus sequence are present.

Sample	Year	Location	412	Blood	Opus	Tirant	Hobo	I-ele	P-ele
SRR23876563	1800	Lund, Sweden	0.82	2.03	0.52	0.12	1.17	8.4	0
SRR23876564	1800	Lund, Sweden	0.68	3.93	1.64	0.14	2.15	9.22	0
SRR23876566	1800	Småland, Sweden	0.57	3.96	3.94	0.1	1.78	9.66	0
SRR23876582	1800	Lund, Sweden	0.95	2.43	0.49	0.16	1.42	8.01	0
SRR23876583	1800	Lund, Sweden	0.55	3.11	0.77	0.11	1.87	7.97	0
SRR23876584	1800	Lund, Sweden	0.85	2.82	0.46	0.12	1.23	7.65	0
SRR23876562	1850	Passau, Germany	0.55	3.41	0.38	0.15	1.88	7.97	0
SRR23876569	1850	Passau, Germany	0.66	5.5	0.44	0.15	2.54	9.43	0
SRR23876565	1875	Zealand, Denmark	17.12	14.67	18.92	4.12	1.74	9.84	0
SRR23876567	1933	Lund, Sweden	41.65	23.89	37.73	0.39	1.59	8.01	0
SRR23876568	1933	Lund, Sweden	52.08	28.06	39.39	0.19	1.31	10	0
SRR23876570	1933	Lund, Sweden	48.62	24.01	51.88	0.48	2.51	11.58	0
SRR23876571	1933	Lund, Sweden	46.2	22.9	30.38	0.29	2.32	13.77	0
SRR23876572	1933	Lund, Sweden	37.5	28.72	37.29	9.61	2.6	11.4	0
SRR23876573	1933	Lund, Sweden	64.32	37.22	74.88	0.44	1.71	12.36	0
SRR23876574	1933	Lund, Sweden	49.59	21.03	43.02	0.16	1.21	10.94	0
SRR23876575	1933	Lund, Sweden	48.36	25.1	33.6	0.18	2.26	11.29	0
SRR23876576	1933	Lund, Sweden	45.24	19.19	33.9	0.2	1.32	12	0
SRR23876577	1933	Lund, Sweden	43.07	26.75	42.7	0.27	2.19	11.23	0
SRR23876578	1933	Lund, Sweden	61.01	27.65	51.32	0.21	1.52	11.54	0
SRR23876579	1933	Lund, Sweden	59.49	22.78	39.25	0.2	1.27	13.02	0
SRR23876580	1933	Lund, Sweden	52.27	22.16	55.14	0.19	1.51	10.68	0
SRR23876581	1933	Lund, Sweden	61.07	21.65	40.02	0.16	1.41	10.52	0
SRR23876585	1933	Lund, Sweden	43.24	25.43	41.77	0.18	2.15	9.31	0
SRR23876586	1933	Lund, Sweden	61.87	27.06	37.55	0.17	1.37	11.25	0
SRR11846555	1936	Crimea, Ukraine	46.74	32.89	30.62	0.65	4.02	16.45	0
SRR11846562	1938	Illinois, USA	39.23	24.55	39.59	2.82	4.94	19.6	0
SRR11846563	1938	Stockholm, Sweden	41.33	25.64	39.4	0.69	23.2	35.32	0.27
SRR11846564	1938	Wisconsin, USA	39.09	19.05	35.01	0.7	3.29	16.89	0
SRR11846556	1950	Berlin, Germany	34.9	17.91	26.61	7.46	3.16	30	0
SRR11846554	1952	Florida, USA	38.18	18.12	34.26	8.75	20.21	29.16	42.71
SRR11846561	1954	Israel	46.97	33.34	25.89	12.29	15.57	38.11	0
SRR11846565	1959	Japan	41.15	12.45	28.95	6.84	5.14	16.05	0
SRR11846560	1967	Massachussets, USA	42.01	16.06	33.49	5.33	10.68	53.68	47.55
SRR11846559	1987	-	58.37	21.73	31.75	3.83	35.68	38.70	0

Table 2: Overview of the GDL strains analysed in this work. For each strain we show the accession number and the location of collection.

**Run accession**	**Location**	**Run accession**	**Location**
SRR1662283	Beijing, China	SRR1663570	Netherlands
SRR1663528	Beijing, China	SRR1663571	Netherlands
SRR1663529	Beijing, China	SRR1663572	Netherlands
SRR1663530	Beijing, China	SRR1663573	Netherlands
SRR1663531	Beijing, China	SRR1663574	Netherlands
SRR1663532	Beijing, China	SRR1663575	Netherlands
SRR1663533	Beijing, China	SRR1663576	Netherlands
SRR1663534	Beijing, China	SRR1663577	Netherlands
SRR1663535	Beijing, China	SRR1663578	Netherlands
SRR1663536	Beijing, China	SRR1663579	Netherlands
SRR1663537	Beijing, China	SRR1663580	Tasmania, Australia
SRR1663538	Beijing, China	SRR1663581	Tasmania, Australia
SRR1663539	Beijing, China	SRR1663582	Tasmania, Australia
SRR1663540	Beijing, China	SRR1663583	Tasmania, Australia
SRR1663541	Beijing, China	SRR1663584	Tasmania, Australia
SRR1663542	Ithaca, USA	SRR1663585	Tasmania, Australia
SRR1663543	Ithaca, USA	SRR1663586	Tasmania, Australia
SRR1663544	Ithaca, USA	SRR1663587	Tasmania, Australia
SRR1663545	Ithaca, USA	SRR1663588	Tasmania, Australia
SRR1663546	Ithaca, USA	SRR1663589	Tasmania, Australia
SRR1663547	Ithaca, USA	SRR1663590	Tasmania, Australia
SRR1663548	Ithaca, USA	SRR1663591	Tasmania, Australia
SRR1663549	Ithaca, USA	SRR1663592	Tasmania, Australia
SRR1663550	Ithaca, USA	SRR1663593	Tasmania, Australia
SRR1663551	Ithaca, USA	SRR1663594	Tasmania, Australia
SRR1663552	Ithaca, USA	SRR1663595	Tasmania, Australia
SRR1663553	Ithaca, USA	SRR1663596	Tasmania, Australia
SRR1663554	Ithaca, USA	SRR1663597	Tasmania, Australia
SRR1663555	Ithaca, USA	SRR1663598	Zimbabwe
SRR1663556	Ithaca, USA	SRR1663599	Zimbabwe
SRR1663557	Ithaca, USA	SRR1663600	Zimbabwe
SRR1663558	Ithaca, USA	SRR1663601	Zimbabwe
SRR1663559	Ithaca, USA	SRR1663602	Zimbabwe
SRR1663560	Ithaca, USA	SRR1663603	Zimbabwe
SRR1663561	Netherlands	SRR1663604	Zimbabwe
SRR1663562	Netherlands	SRR1663605	Zimbabwe
SRR1663563	Netherlands	SRR1663606	Zimbabwe
SRR1663564	Netherlands	SRR1663607	Zimbabwe
SRR1663565	Netherlands	SRR1663608	Zimbabwe
SRR1663566	Netherlands	SRR1663609	Zimbabwe
SRR1663567	Netherlands	SRR1663610	Zimbabwe
SRR1663568	Netherlands	SRR1663611	Zimbabwe
SRR1663569	Netherlands		

Table 3: Overview of the most abundant diagnostic SNPs, for identifying population structure of Tirant, 412 and Opus. For each diagnostic SNP we show the position in the TE (position), the population where the SNP is most abundant (population), a frequency threshold and the abundance of the SNP inside (in) and outside (out) of the given population. For example, the first entry shows that the SNP in Tirant at position 5628 has a frequency of at least 0.5 (threshold) in 68% of the samples from Tasmania (in) but only in 10% of the other samples (i.e. not-Tasmania; out).

**TE**	**position**	**population**	**threshold**	**in**	**out**
Tirant	5628	Ithaca, USA	0.5	68%	10%
Tirant	6757	Ithaca, USA	0.5	63%	19%
Tirant	2254	Ithaca, USA	0.3	63%	0%
Tirant	243	Tasmania, Australia	0.5	94%	20%
Tirant	275	Tasmania, Australia	0.5	100%	11%
Tirant	3921	Tasmania, Australia	0.5	94%	0%
Tirant	4374	Tasmania, Australia	0.5	83%	0%
Tirant	8350	Tasmania, Australia	0.5	100%	16%
Tirant	8382	Tasmania, Australia	0.5	100%	9%
Tirant	5091	Tasmania, Australia	0.4	72%	0%
Tirant	3733	Beijing, China	0.4	60%	7%
Tirant	1547	Beijing, China	0.3	80%	3%
Tirant	4311	Netherlands	0.3	58%	20%
412	7199	Zimbabwe	0.5	79%	4%
412	7168	Zimbabwe	0.4	64%	0%
412	115	Zimbabwe	0.3	79%	3%
412	1530	Zimbabwe	0.3	71%	6%
412	1536	Zimbabwe	0.3	71%	7%
412	1542	Zimbabwe	0.3	71%	7%
412	1554	Zimbabwe	0.3	57%	6%
412	369	Beijing, China	0.4	53%	11%
412	5700	Beijing, China	0.4	60%	11%
412	7422	Beijing, China	0.4	60%	13%
Opus	325	Tasmania, Australia	0.5	78%	6%
Opus	7328	Tasmania, Australia	0.5	78%	7%
Opus	7369	Zimbabwe	0.5	71%	0%
Opus	366	Zimbabwe	0.4	79%	0%
Opus	6385	Ithaca, USA	0.4	84%	20%

Table 4: TE content in four long-read assemblies of *D. melanogaster*. For each of the seven TEs that invaded *D. melanogaster* populations during the last 200 years we show the genomic proportion in kbp. The assembly size (size) and the genomic proportion occupied by these seven TEs is also shown.

	Pi2	DGRP-732	Iso1	Canton-S
size	167,834	141,551	143,726	149,105
412	312	234	242	249
Blood	260	99	218	261
Opus	263	167	248	221
Tirant	69	84	161	0
I-element	81	123	68	0
Hobo	188	55	33	0
P-element	58	41	0	0
Sum TEs	1,230	803	970	731
proportion [%]	0.73	0.57	0.68	0.49

Table 5: Overview of the long-read assemblies of diverse insect species analysed in this work.

order	family	genus	taxon	accession
Coleoptera	Coccinellidae	Cryptolaemus	Cryptolaemus.montrouzieri	GCA_013387265.1
Coleoptera	Coccinellidae	Harmonia	Harmonia.axyridis	GCA_011033045.1
Coleoptera	Coccinellidae	Propylea	Propylea.japonica	GCA_013421045.1
Coleoptera	Curculionidae	Listronotus	Listronotus.bonariensis	GCA_014170235.1
Coleoptera	Curculionidae	Sitophilus	Sitophilus.oryzae	GCA_002938485.2
Coleoptera	Elateridae	Limonius	Limonius.californicus	GCA_014611495.1
Coleoptera	Lampyridae	Abscondita	Abscondita.terminalis	GCA_013368085.1
Coleoptera	Lampyridae	Lamprigera	Lamprigera.yunnana	GCA_013368075.1
Coleoptera	Lampyridae	Photinus	Photinus.pyralis	GCA_008802855.1
Coleoptera	Nitidulidae	Aethina	Aethina.tumida	GCA_001937115.1
Coleoptera	Scarabaeidae	Protaetia	Protaetia.brevitarsis	GCA_004143645.1
Coleoptera	Scarabaeidae	Trypoxylus	Trypoxylus.dichotomus	GCA_014905495.1
Coleoptera	Silphidae	Nicrophorus	Nicrophorus.vespilloides	GCA_001412225.1
Collembola	Entomobryidae	Sinella	Sinella.curviseta	GCA_004115045.2
Collembola	Isotomidae	Folsomia	Folsomia.candida	GCA_002217175.1
Collembola	Orchesellidae	Orchesella	Orchesella.cincta	GCA_001718145.1
Diptera	Calliphoridae	Cochliomyia	Cochliomyia.hominivorax	GCA_004302925.1
Diptera	Calliphoridae	Phormia	Phormia.regina	GCA_001735545.1
Diptera	Chironomidae	Belgica	Belgica.antarctica	GCA_000775305.1
Diptera	Culicidae	Aedes	Aedes.albopictus	GCA_001876365.2
Diptera	Culicidae	Aedes	Aedes.aegypti	GCA_002204515.1
Diptera	Culicidae	Anopheles	Anopheles.albimanus	GCA_013758885.1
Diptera	Culicidae	Anopheles	Anopheles.gambiae	GCA_001542645.1
Diptera	Culicidae	Anopheles	Anopheles.funestus	GCA_003951495.1
Diptera	Culicidae	Anopheles	Anopheles.stephensi	GCA_013141755.1
Diptera	Culicidae	Anopheles	Anopheles.coluzzii	GCA_004136515.2
Diptera	Diopsidae	Teleopsis	Teleopsis.dalmanni	GCA_002237135.1
Diptera	Muscidae	Haematobia	Haematobia.irritans	GCA_003123925.1
Diptera	Muscidae	Musca	Musca.domestica	GCA_014843735.1
Diptera	Sarcophagidae	Sarcophaga	Sarcophaga.peregrina	GCA_014635995.1
Diptera	Sciaridae	Bradysia	Bradysia.coprophila	GCA_014529535.1
Diptera	Tephritidae	Bactrocera	Bactrocera.oleae	GCA_001188975.4
Hemiptera	Aleyrodidae	Bemisia	Bemisia.tabaci	GCA_001854935.1
Hemiptera	Aleyrodidae	Trialeurodes	Trialeurodes.vaporariorum	GCA_011764245.1
Hemiptera	Anthocoridae	Orius	Orius.insidiosus	GCA_014119065.1
Hemiptera	Aphididae	Aphis	Aphis.glycines	GCA_009928515.1
Hemiptera	Aphididae	Rhopalosiphum	Rhopalosiphum.maidis	GCA_003676215.3
Hemiptera	Aphididae	Sitobion	Sitobion.miscanthi	GCA_008086715.1
Hemiptera	Delphacidae	Laodelphax	Laodelphax.striatellus	GCA_003335185.2
Hemiptera	Delphacidae	Nilaparvata	Nilaparvata.lugens	GCA_014356525.1
Hemiptera	Liviidae	Diaphorina	Diaphorina.citri	GCA_000475195.1
Hemiptera	Miridae	Apolygus	Apolygus.lucorum	GCA_009739505.2
Hemiptera	Pentatomidae	Euschistus	Euschistus.heros	GCA_003667255.1
Hemiptera	Pseudococcidae	Maconellicoccus	Maconellicoccus.hirsutus	GCA_003261595.1
Hemiptera	Pseudococcidae	Phenacoccus	Phenacoccus.solenopsis	GCA_009761765.1
Hemiptera	Reduviidae	Triatoma	Triatoma.infestans	GCA_011037195.1
Hymenoptera	Apidae	Apis	Apis.mellifera	GCA_003254395.2
Hymenoptera	Apidae	Apis	Apis.dorsata	GCA_009792835.1
Hymenoptera	Apidae	Apis	Apis.mellifera	GCA_003314205.1
Hymenoptera	Apidae	Apis	Apis.cerana	GCA_011100585.1
Hymenoptera	Apidae	Apis	Apis.mellifera	GCA_013841205.1
Hymenoptera	Apidae	Apis	Apis.mellifera	GCA_013841245.1
Hymenoptera	Apidae	Bombus	Bombus.vosnesenskii	GCA_011952255.1
Hymenoptera	Apidae	Bombus	Bombus.bifarius	GCA_011952205.1
Hymenoptera	Apidae	Bombus	Bombus.vancouverensis	GCA_011952275.1
Hymenoptera	Braconidae	Aphidius	Aphidius.gifuensis	GCA_014905175.1
Hymenoptera	Braconidae	Aphidius	Aphidius.ervi	GCA_011426455.1
Hymenoptera	Braconidae	Chelonus	Chelonus.insularis	GCA_013357705.1
Hymenoptera	Braconidae	Lysiphlebus	Lysiphlebus.fabarum	GCA_011426435.1
Hymenoptera	Colletidae	Colletes	Colletes.gigas	GCA_013123115.1
Hymenoptera	Figitidae	Leptopilina	Leptopilina.clavipes	GCA_001855655.1
Hymenoptera	Figitidae	Leptopilina	Leptopilina.boulardi	GCA_011634795.1
Hymenoptera	Formicidae	Camponotus	Camponotus.floridanus	GCA_003227725.1
Hymenoptera	Formicidae	Formica	Formica.selysi	GCA_009859135.1
Hymenoptera	Formicidae	Harpegnathos	Harpegnathos.saltator	GCA_003227715.1
Hymenoptera	Formicidae	Monomorium	Monomorium.pharaonis	GCA_013373865.2
Hymenoptera	Formicidae	Nylanderia	Nylanderia.fulva	GCA_005281655.1
Hymenoptera	Formicidae	Ooceraea	Ooceraea.biroi	GCA_003672135.1
Hymenoptera	Formicidae	Solenopsis	Solenopsis.invicta	GCA_010367695.1
Hymenoptera	Megachilidae	Osmia	Osmia.lignaria	GCA_012274295.1
Hymenoptera	Pteromalidae	Nasonia	Nasonia.vitripennis	GCA_009193385.2
Hymenoptera	Pteromalidae	Pteromalus	Pteromalus.puparum	GCA_012977825.2
Hymenoptera	Vespidae	Polistes	Polistes.metricus	GCA_010416925.1
Hymenoptera	Vespidae	Polistes	Polistes.fuscatus	GCA_010416935.1
Hymenoptera	Vespidae	Vespa	Vespa.mandarinia	GCA_014083535.1
Lepidoptera	Carposinidae	Carposina	Carposina.sasakii	GCA_014607495.2
Lepidoptera	Crambidae	Chilo	Chilo.suppressalis	GCA_004000445.1
Lepidoptera	Crambidae	Cnaphalocrocis	Cnaphalocrocis.medinalis	GCA_014851415.1
Lepidoptera	Hesperiidae	Epargyreus	Epargyreus.clarus	GCA_014595695.1
Lepidoptera	Lasiocampidae	Dendrolimus	Dendrolimus.punctatus	GCA_012273795.1
Lepidoptera	Noctuidae	Heliothis	Heliothis.virescens	GCA_002382865.1
Lepidoptera	Noctuidae	Spodoptera	Spodoptera.exigua	GCA_011316535.1
Lepidoptera	Noctuidae	Spodoptera	Spodoptera.frugiperda	GCA_011064685.1
Lepidoptera	Noctuidae	Trichoplusia	Trichoplusia.ni	GCA_003590095.1
Lepidoptera	Nymphalidae	Maniola	Maniola.jurtina	GCA_009667785.1
Lepidoptera	Pieridae	Colias	Colias.croceus	GCA_009982905.1
Lepidoptera	Psychidae	Eumeta	Eumeta.japonica	GCA_005406025.1
Lepidoptera	Pyralidae	Galleria	Galleria.mellonella	GCA_004355975.1
Lepidoptera	Saturniidae	Antheraea	Antheraea.mylitta	GCA_014332785.1
Lepidoptera	Saturniidae	Samia	Samia.ricini	GCA_014132275.1
Lepidoptera	Sphingidae	Hyles	Hyles.vespertilio	GCA_009982885.1
Lepidoptera	Sphingidae	Manduca	Manduca.sexta	GCA_014839805.1
Lepidoptera	Tortricidae	Cydia	Cydia.pomonella	GCA_003425675.2
Orthoptera	Gryllidae	Teleogryllus	Teleogryllus.occipitalis	GCA_011170035.1
Siphonaptera	Pulicidae	Ctenocephalides	Ctenocephalides.felis	GCA_003426905.1
Thysanoptera	Thripidae	Thrips	Thrips.palmi	GCA_012932325.1
Trichoptera	Hydropsychidae	Hydropsyche	Hydropsyche.tenuis	GCA_009617725.1
Trichoptera	Polycentropodidae	Plectrocnemia	Plectrocnemia.conspersa	GCA_009617715.1
Trichoptera	Stenopsychidae	Stenopsyche	Stenopsyche.tienmushanensis	GCA_008973525.1

References

C. M. Bergman and D. Bensasson. Recent LTR retrotransposon insertion contrasts with waves of non-LTR insertion since speciation in *Drosophila melanogaster*. *PNAS*, 104(27):11340–5, July 2007.

N. J. Bowen and J. F. McDonald. Drosophila euchromatic LTR retrotransposons are much younger than the host species in which they reside. *Genome research*, 11(9):1527–1540, 2001.

M. G. Kidwell. Evolution of hybrid dysgenesis determinants in *Drosophila melanogaster*. *Proceedings of the National Academy of Sciences*, 80(6):1655–1659, 1983.

S. Luo, H. Zhang, Y. Duan, X. Yao, A. G. Clark, and J. Lu. The evolutionary arms race between transposable elements and piRNAs in *Drosophila melanogaster*. *BMC Evolutionary Biology*, 20(1):1–18, 2020.

F. Schwarz, F. Wierzbicki, K.-A. Senti, and R. Kofler. Tirant stealthily invaded natural drosophila melanogaster populations during the last century. *Molecular Biology and Evolution*, 38(4):1482–1497, 2021.

K. Tamura, S. Subramanian, and S. Kumar. Temporal Patterns of Fruit Fly (Drosophila) Evolution Revealed by Mutation Clocks. *Molecular Biology and Evolution*, 21(1):36–44, 01 2004.

L. Weilguny and R. Kofler. DeviaTE: Assembly-free analysis and visualization of mobile genetic element composition. *Molecular ecology resources*, 19(5):1346–1354, 2019.
